# Dnmt3a-mediated *de novo* methylation balances memory Th1 and Tfh cell plasticity and functionality

**DOI:** 10.1101/2024.12.03.623450

**Published:** 2024-12-06

**Authors:** Bryant Perkins, Camille Novis, Andrew Baessler, Linda M Sircy, Monyca M Thomas, Malia Harrison-Chau, Andrew W Richens, Bryce Fuchs, Nguyen X. Nguyen, Kaitlyn Flint, Katherine E Varley, J. Scott Hale

**Affiliations:** 1 Division of Microbiology and Immunology, Department of Pathology, University of Utah School of Medicine, Salt Lake City, UT 84112; 2 Department of Oncological Sciences, Huntsman Cancer Institute, University of Utah School of Medicine, Salt Lake City, UT 84112, USA

## Abstract

Following acute viral infection, naïve CD4+ T cells differentiate into T follicular helper (Tfh) and T helper 1 (Th1) cells that generate long-lived memory cells. However, it is unclear how memory Tfh and Th1 cells maintain their lineage commitment. Here we demonstrate that Tfh and Th1 lineages acquire distinct *de novo* DNA methylation programs that are preserved into memory. Using whole genome methylation analyses and deletion of DNA methyltransferase 3a, we found that *de novo* DNA methylation is required for generating epigenetic programing to enforce lineage commitment and preserve lineage-specific functions during a recall response to infection. Importantly, partial inhibition of *de novo* methylation using the methyltransferase inhibitor decitabine during priming enhances Tfh cell functionality in primary and secondary responses to viral infection. Together, these findings demonstrate that *de novo* DNA methylation is critical to balance lineage commitment and functionality of memory CD4+ T cell subsets and reveal novel potential strategies to modulate immune responses to infectious diseases.

## SUMMARY

Effector Tfh and Th1 cells acquire lineage-specific *de novo* DNA methylation programs that are required to enforce lineage commitment and preserve functionality of memory Tfh and Th1 cells during the recall response to viral infection.

## INTRODUCTION

In response to acute viral infections, the immune system utilizes humoral and cellular mediated mechanisms to clear infections and provide lasting protection against future infection. CD4+ T helper cells are critical in both aspects. Naïve CD4+ T helper cells differentiate into T helper 1 (Th1) and T follicular helper (Tfh) cells upon encountering viral pathogens. Th1 cells migrate to sites of infection where they play a critical role in coordinating cell-mediated immune response^1^. Tfh cells are critical regulators of the humoral immune response that are required for formation of the germinal center (GC), a transient substructure of the B cell follicle where GC B cells undergo clonal selection, affinity maturation, and differentiation into memory B cells and plasma cells^2^. Following antigen clearance, the majority of effector T helper cells undergo apoptosis, leaving behind a population T helper cells persist to form long-lived memory^3^. Both Tfh and Th1 cells have the capacity to form long-lived memory cells that preferentially recall their specialized effector functions upon viral reinfection^4–15^. Thus, Tfh and Th1 cells are not only important for controlling ongoing infections, but also for providing long term protection.

T helper cell differentiation is driven by environmental signals that orchestrate cellular reprograming. Intrinsically, a complex network of transcription factors determines the Tfh or Th1 cell fate decision. Tfh cells are defined by expression of Bcl6, while Th1 cells express Tbet. Additional factors include the Tfh promoting factors Tcf1 and Lef1, as well as the Tfh repressors Blimp1, Foxo1, Runx2 and Runx3^16^. Mechanistically, transcription factors coordinate the epigenetic reprograming of the cell to drive differentiation and establish heritable transcriptional programing. DNA methylation is a type of epigenetic modification where methyl groups are added to CpG dinucleotides within the genome to silence gene expression^17^. T helper differentiation is accompanied by changes in DNA methylation^5, 6, 18–27^ and these changes are maintained by Dnmt1 during replication^17^. Research has demonstrated that changes in DNA methylation regulates T helper differentiation and function^5, 6, 18–27^. For example, the demethylating enzyme Tet2 inhibits GC Tfh cell differentiation during viral infection through coordination with the Tfh repressors Foxo1 and Runx1^18^. The DNA methyltransferase Dnmt3a catalyzes *de novo* methylation in CD4+ T cells^25, 26^. Dnmt3a regulates T helper functionality by silencing IFNγ production in non-Th1 cells^25, 26^, and restraining Il-13 production in Th2 cells^23^. However, the role of Dnmt3a in Tfh differentiation and function is unknown. Another important role for DNA methylation is to regulate the lineage commitment of memory T helper cells, which no longer express key lineage-defining and activation-associated transcription factors, such as Bcl6 and Blimp1^5^. Deletion of Tet2 enhanced the plasticity of virus-specific memory Th1 cells during the recall response^6^. However, whether Dnmt3a regulates the memory Tfh and Th1 cell formation and lineage commitment is unknown.

In this study, we examined the role of Dnmt3a and *de novo* methylation programing in T helper cell differentiation, memory formation, and lineage commitment during viral infection. We report that the Tfh and Th1 lineages acquire distinct *de novo* methylation programs that are mediated by Dnmt3a and that are maintained in memory Tfh and Th1 cells. Loss of Dnmt3a-mediated programing by genetic deletion or reduced Dnmt3a activity via transient pharmaceutical inhibition using decitabine early during T cell priming enhanced the primary GC Tfh cell response and impaired the lineage commitment of memory Th1 cells. We partially attribute the enhanced plasticity and increased GC Tfh potential of memory Th1 cells to a failure to silence *Tcf7* and *Lef1*. However, deletion of Dnmt3a drastically impaired Tfh and Th1 cell functionality during the memory recall response, suggesting that Dnmt3a regulates functionality at the expense of plasticity. Importantly, partial inhibition of Dnmt3a via decitabine treatment during priming enhanced both the plasticity and functionality of the memory recall response to viral infection. Therefore, our findings demonstrate that Dnmt3a-mediated *de novo* methylation programing is a targetable pathway that fine tunes the balance between lineage plasticity and functionality of the memory CD4+ T cell recall response to viral infections.

## RESULTS

### Lineage-specific de novo methylation programing is maintained in memory Tfh and Th1 cells

**Figure 1.**
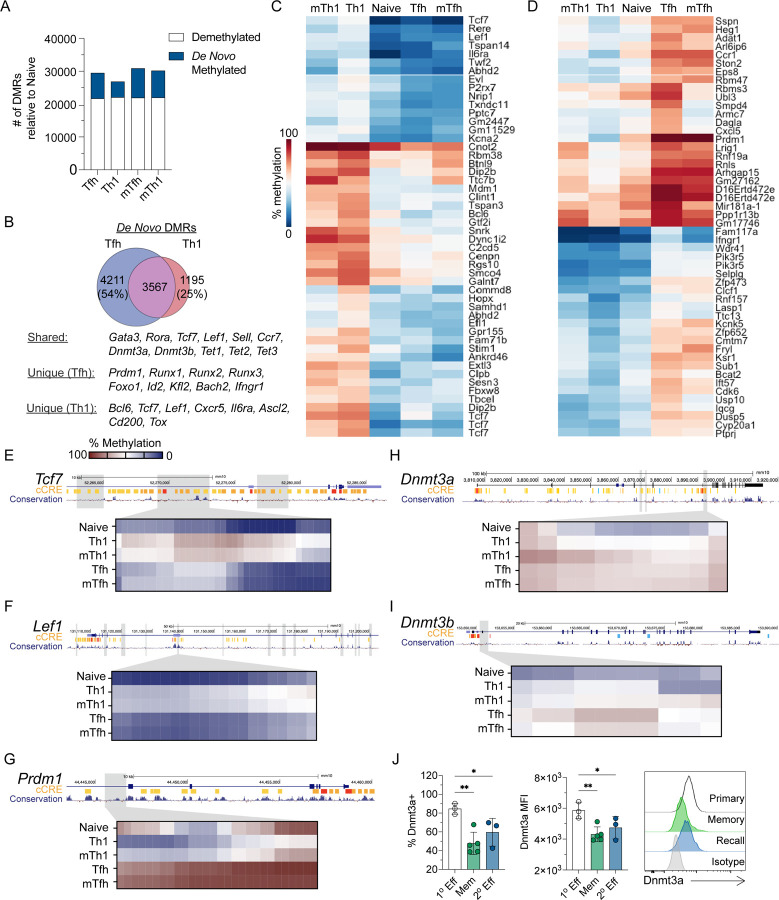
Lineage-specific *de novo* methylation programing is maintained in memory Tfh and Th1 cells. Whole genome bisulfite sequencing was performed on DNA isolated from Tfh and Th1 cells sorted at effector (Day 7) or memory timepoints (Day 60+) following LCMV infection. DMRs were identified between each cell type relative to Naïve CD4+ T cells. (**A**) Total number of DMRs identified for each cell type, broken down as *de novo* methylated or demethylated relative to Naïve CD4+ T cells. (**B**) Venn diagram of overlapping (shared) *de novo* DMRs between effector Tfh and Th1. (C and D) Heatmaps of the top 50 lineage-specific *de novo* DMRs for (**C**) Th1 or (**D**) Tfh cells. DMRs were ranked by mean difference in methylation between Tfh and Th1 cells. (E-I) UCSC genome browser snapshots of (**E**) *Tcf7*, (**F**) *Lef1*, (**G**) *Prdm1*, (**H**) *Dnmt3a*, and (**I**) *Dnmt3b*. Tracks show ENCODE identified candidate cis regulatory elements (cCREs) and mammalian conservation. Grey boxes identify *de novo* DMRs. Select DMRs are highlighted in the heatmaps below and each column represents a single CpG site within the region. (**J**) SMARTA cells were adoptively transferred into B6 mice and infected with LCMV. Flow cytometric analysis was performed on primary effector cells (Day 7), memory cells (Day 42), or secondary effector cells (Day 7). Frequency of Dnmt3a+ SMARTA cells and Dnmt3a MFI of SMARTA cells are shown as are representative Dnmt3a histograms. Significant p values (* p<0.05, **p<0.01) were determined by one-way ANOVA.

To address whether *de novo* methylation programing underpins effector and memory Tfh and Th1 differentiation, we used the acute lymphocytic choriomeningitis virus (LCMV) infection model. Naive SMARTA TCR transgenic CD4+ T cells (CD45.1+) that are specific for the LCMV GP^66−77^ epitope were adoptively transferred into C57BL/6J (B6) mice^28^. Recipient mice were subsequently infected with LCMV. At 7 and 70+ days post infection (dpi), Tfh (CXCR5+ Ly6C^LO^) and Th1 (CXCR5− Ly6C^HI^) cells were sorted to isolate genomic DNA for whole genome DNA methylation analysis. Differentially methylated regions (DMRs) were identified for effector or memory Tfh and Th1 cells relative to naïve T cells. This analysis revealed genome wide changes in methylation programing at over 25k regions per cell type relative to Naïve CD4+ T cell (Fig. 1A). *De novo* methylation accounted for a small fraction of these DMRs (Fig. 1A).

During LCMV infection, Tfh cells partially take on Th1 cell transcriptional qualities, including intermediate expression of Tbet^5^. Therefore, we reasoned that aspects of *de novo* methylation programing may be shared and that the unique programing would be foundational to Tfh and Th1 cell identity and lineage commitment. To determine shared *de novo* methylation programs of Tfh and Th1 effector cells, we first identified overlapping DMRs. Tfh cells shared 46% of *de novo* DMRs with Th1 cells, while 75% of Th1 *de novo* DMRs were shared with Tfh cells (Fig. 1B). Shared *de novo* DMRs were found at genes associated with Th2/Th17 cells (*Gata3, Rora*), memory formation (*Sell, Ccr7, Bcl2*), and DNA methylation (*Dnmt3a, Dnmt3b, Tet2*). We next ranked *de novo* DMRs based on mean difference in methylation between Tfh and Th1 cells. Our heatmap analysis of the top 50 Th1-specific DMRs highlighted several Tfh genes which acquired *de novo* methylation (*Tcf7, Lef1, Bcl6*, *P2rx7*, and *Il6ra*) (Fig. 1C). The top 50 Tfh-specific *de novo* DMRs included the Th1 genes *Prdm1, Ifngr1*, and *Selplg* (Fig. 1D). We further analyzed the genomic locations of select Th1 DMRs and found evidence of extensive intragenic *de novo* methylation at *Tcf7* and *Lef1* that overlapped with regions of mammalian conservation (Fig. 1E–F). In Tfh cells, we found clear evidence of *de novo* methylation within the *Prdm1* locus (Fig. 1G). Of note, this Tfh *de novo* DMR underwent demethylation in the Th1 lineage which express Blimp1. Overall, these analyses demonstrates that effector and memory Tfh and Th1 cells acquire unique *de novo* methylation programing which may be linked to lineage commitment.

These analyses also revealed that the *de novo* methylation programs were shared between effector and memory T cells of the same lineage (Fig. 1C–I), indicating that lineage-specific *de novo* DNA methylation programing was acquired during T cell differentiation during the primary response and then maintained into memory. In agreement with this idea, we observed *de novo* DMRs at the genes *Dnmt3a* and *Dnmt3b* (Fig. 1H–I). To determine whether *de novo* methylation at the *Dnmt3a* locus correlated with reduced expression in memory and secondary (2°) effector T cells, we measured Dnmt3a by flow cytometry. For primary effector or memory timepoints, SMARTA T cells were adoptively transferred into B6 mice and infected with LCMV. For memory recall experiments, SMARTA T cells were enriched at memory timepoint and transferred into naïve B6 recipients, which were subsequently infected with LCMV. At the primary effector timepoint (7 dpi), we found that greater than 80% of SMARTA T cells expressed Dnmt3a (Fig 1J). In contrast, there was a significant reduction in the percent of SMARTA cells which were Dnmt3a+, and the MFI was reduced at memory and recall timepoints (Fig. 1J). These data imply that *de novo* methylation programing is largely acquired during the primary infection when Tfh and Th1 lineage commitment occurs^15^.

### Early decitabine treatment during T cell priming enhances germinal center Tfh differentiation

**Figure 2.**
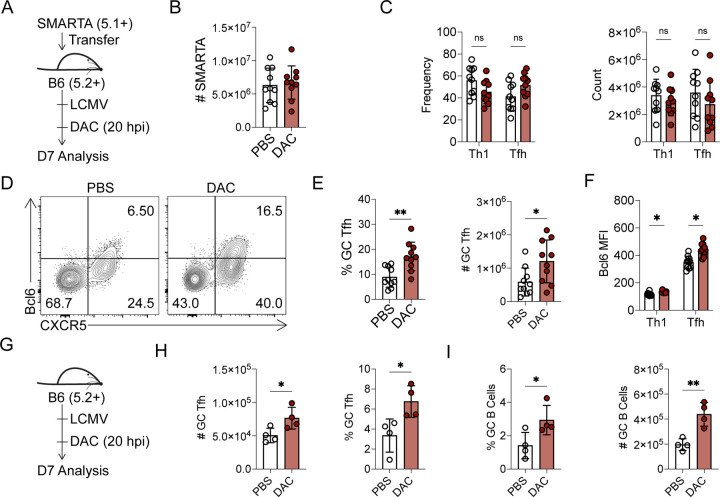
Early decitabine treatment during T cell priming enhances germinal center Tfh differentiation. (A-F) Naïve SMARTA cells were transferred into B6 mice which were subsequently infected with LCMV. At 20 hours post infection, mice received an IP injection of either DAC (0.75 mg/kg) or PBS. Analysis was performed on splenocytes at 7 days post infection. (**A**) experimental schematic. (**B**) number of SMARTA cells. (**C**) Flow cytometry plot of CXCR5 and Bcl6 for identification of GC Tfh, gated on SMARTA T cells. (**D**) Percent and number of CXCR5+ Tfh cells, CXCR5− Tfh cells, or (**E**) CXCR5+ Bcl6^HI^ GC Tfh SMARTA cells. (**F**) Bcl6 MFI of Tfh and Th1 SMARTA cells. (G-H) In a follow up experiment, B6 mice were infected with LCMV and given either a single dose of DAC or PBS at 20 hours post infection. (**G**) FACS plot of GL7 and FAS, gated on B cells, for identification of GC B Cells. (**H**) Percent and number of PD-1^HI^ GC Tfh cells and (**I**) GC B cells. Data are representative of three or more independent experiments. * p<0.05, **p<0.01. Statistical significance was determined by a student’s t test.

Given the unique *de novo* methylation programing that are acquired during Tfh and Th1 cell differentiation, we reasoned that inhibition of *de novo* methylation programing would alter T helper differentiation and function. To address this, we first used an inhibitor of DNA methyltransferases, Decitabine (5’-Aza-2’-deoxycitidine; herein DAC)^29–31^. Dnmt3a is upregulated within 24 hours *in vitro*^25, 26^, so we reasoned that DAC treatment would need to occur early to inhibit *de novo* methylation. DAC is also cytotoxic at high concentrations^29–31^. Therefore, we first performed *in vivo* titrations of DAC to find a dose and treatment window that could alter CD4+ T cell differentiation without impairing T and B cell responses (Fig. S1A). At doses of 0.75 mg/kg, we found no difference in the number of B cells or T cells relative to PBS control (Fig S1B–F), suggesting this dose did not induce cell death. Intriguingly, we did find evidence of increased Bcl6 expression in Tfh cells, even at higher cytotoxic doses (Fig. S1G–H). In follow up experiments, naïve SMARTA cells were transferred into B6 recipient mice, infected with LCMV, and treated at 20 hours post infection (hpi) with either DAC (0.75mg/kg) or PBS (Fig. 2A). DAC did not alter SMARTA T effector cell accumulation or differentiation (Fig. 2B–C). However, DAC treatment significantly increased the percent and number of Bcl6^HI^ GC Tfh cells (Fig. 2D–E) as well as the Bcl6 MFI in Tfh/Th1 cells (Fig. 2F). Thus, early decitabine treatment during T cell priming enhanced Bcl6 expression and GC Tfh cell differentiation.

The Blimp1-Bcl6 axis is one key pathway that regulates GC Tfh cell differentiation^32–39^. Therefore, we next asked if DAC treatment altered the Blimp1-Bcl6 axis at 3 dpi. Naïve SMARTA cells were transferred into B6 recipients, which were intravenously infected with LCMV (Fig. S1I). At 3 dpi, we found no difference in the number of SMARTA cells (Fig. S1J), or in the number of early Th1/Tfh cells (Fig. S1K). However, we did find enhanced Bcl6 expression in both Tfh and Th1 cells (Fig. S1L). This correlated with a trending reduction in Blimp1 expression in Tfh (p=0.058) and Th1 (p=0.067) cells (Fig. S1M). These data indicate that DAC enhanced Bcl6 expression while having minimal effect on early cell fate decisions.

To determine whether DAC enhanced the polyclonal T cell response to LCMV infection, B6 mice were infected with LCMV and treated with DAC at 20 hpi (Fig. 2G). As in the TCR transgenic model (Fig. 2E), we observed evidence of enhanced PD-1^HI^ GC Tfh cell differentiation (Fig. 2H), as well as increased GC B cell responses following DAC treatment (Fig 2I). These data indicate that early DAC treatment enhanced polyclonal GC Tfh differentiation and help for GC B cells during viral infection.

### Decitabine treatment during T cell priming enhances the polyclonal GC Tfh response to influenza infection

**Figure 3.**
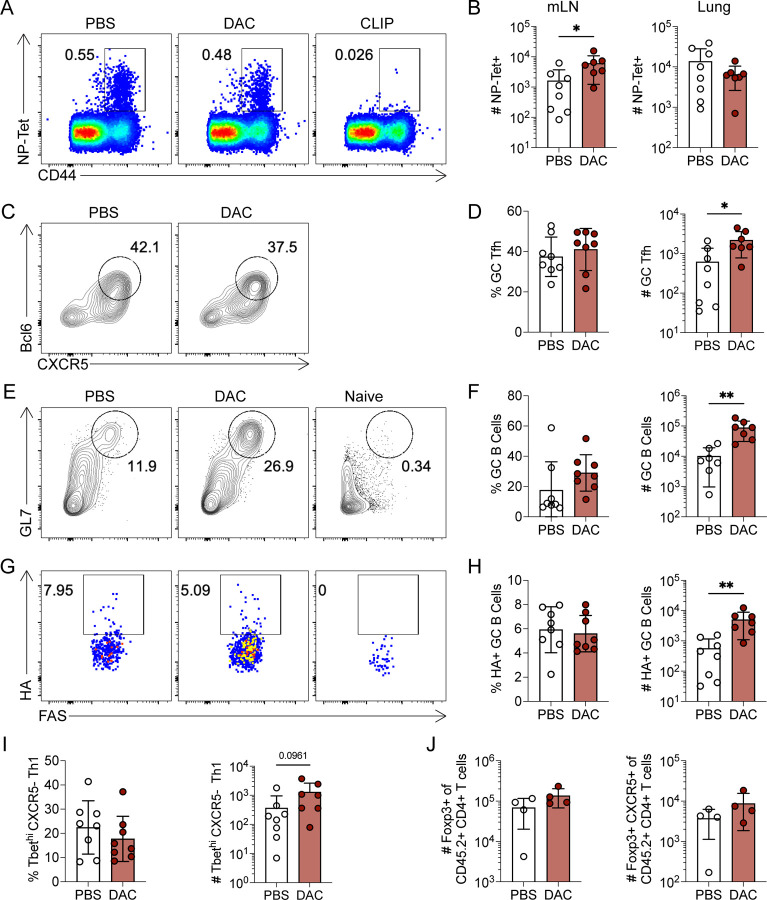
Decitabine treatment during T cell priming enhances the polyclonal GC Tfh response to influenza infection. (A-H) B6 mice were intranasally infected with the murine adapted influenza strain PR8. Mice were treated with DAC (0.375 mg/kg) or PBS at 20 hours post infection. Mediastinal lymph nodes (mLNs) and lung responses were analyzed at 8 days post infection. (**A**) FACS plots of NP-tetramer and CD44 staining, for identification of flu-specific CD4+ T cells. (**B**) Number of NP-tetramer specific CD4+ T cells in the mLNs or lung tissues. (**C**) FACS plots of CXCR5 and Bcl6 for GC Tfh identification, gated on tetramer positive CD4+ T cells. (**D**) Percent and number of CXCR5+ Bcl6^HI^ GC Tfh cells. (**E**) FACS plots of GL7 and FAS for GC B Cell identification, gated on IgD- B cells. (**F**) Percent and number of GC B cells. (**G**) FACS plots of HA-probe and FAS for identification of flu-specific GC B cells, gated on GC B cells. (**H**) Percent and numbers of flu specific GC B Cells. (**I**) Quantification of the percent and number of Tbet^HI^ CXCR5− Th1 cells in the mLNs. (**J**) Quantification of the percent and number of Foxp3+ Treg cells in the mLNs. Mice were excluded from this experiment based on the following criteria: the mice did not lose weight and lacked detectable antigen specific CD4+ T cells in the lung and mLNs. Data are representative of two independent experiments. * p<0.05, **p<0.01. Statistical significance was determined by a student’s t test.

We next asked whether DAC treatment could enhance GC Tfh cell differentiation in other viral infections. The influenza model PR8 was chosen because it would enable us to track antigen specific T and B cell responses. Briefly, B6 mice were intranasally infected with 200 TCID_50_ of PR8 and treated with DAC (0.375 mg/kg) at 20 hpi. A lower dose of DAC was used because we observed signs of cytotoxicity at the normal dose in this model (data not shown). We analyzed the T cell response at 8 dpi and found a significant increase in NP-specific CD4+ T cells in the mediastinal lymph nodes (mLNs) but not the lung of DAC treated recipients (Fig. 3A–B). In the mLNs of DAC treated recipients, we observed a significant increase in the number of Bcl6^HI^ GC Tfh cells (Fig 3C–D). We also observed a significant increase in the number of GC B cells (Fig 3E–F) and hemagglutinin-specific GC B cells (Fig. 3G–H). We found no difference in the Th1 response in the mLNs (Fig. 3I). One possible explanation for the enhanced GC Tfh cell response is that DAC treatment may alter regulatory T cell responses. However, we observed no difference in either CXCR5− or CXCR5+ regulatory T cells in the mLNs (Fig. 3J). Combined, these data indicate that early DAC treatment enhanced GC Tfh differentiation and function to multiple viral infections.

### Decitabine treatment during T cell priming enhances memory T cell formation

**Figure 4.**
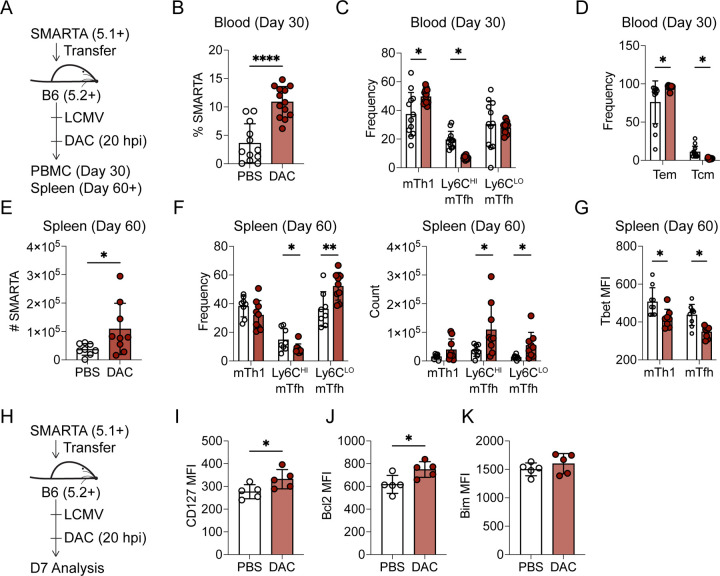
Decitabine treatment during T cell priming enhances memory T cell formation. (A-G) Naïve SMARTA cells were transferred into B6 mice which were subsequently infected with LCMV. At 20 hours post infection, mice received an IP injection of either DAC (0.75 mg/kg) or PBS. (**A**) Experimental schematic. (B-D) Data was analyzed 30 days post infection from blood. (**B**) Percent of SMARTA memory T cells in PBMC. (**C**) Percent of CXCR5− Ly6C^HI^ memory Th1, CXCR5+ Ly6C^HI^ memory Tfh or CXCR5+ Ly6C^LO^ memory Tfh cells in PBMC. (**D**) Percent CD44+ CD62L- T effector memory (Tem) or CD44+ CD62L+ T central memory (Tcm). Data is representative of three independent experiments. (E-G) Analysis of SMARTA cells 60+ days post infection in the spleen. (**E**) Number of SMARTA cells in the spleen. (**F**) Frequency and number of CXCR5− Ly6C^HI^ memory Th1, CXCR5+ Ly6C^HI^ memory Tfh or CXCR5+ Ly6C^LO^ memory Tfh cells in the spleen. (**G**) Tbet MFI of memory Tfh or Th1 cells in the spleen. Data is representative of three independent experiments. (H-K) Analysis of survival markers at 7 days post infection. (**H**) Experimental schematic. MFI of (**I**) CD127, (**J**) Bcl2, or (**K**) Bim gated on SMARTA cells at 7 days post infection. Data are representative of three or more independent experiments. * p<0.05, **p<0.01. Statistical significance was determined by a student’s t test.

We next examined the effects of DAC treatment on memory cell formation (Fig 4A). We analyzed circulating memory cells in PBMC and found a significant increase in the frequency of SMARTA memory T cells at day 30 in DAC treated recipients (Fig. 4B). Phenotypically, there was a small but significant increase in the fraction of memory Th1 (mTh1) cells (Fig. 4C) and in T effector memory cells (Fig. 4D). Similarly, we observed a significant increase in the number of SMARTA memory T cells in the spleen at 70 dpi (Fig 4E). In contrast to the blood, we found an increase in the percent and number of Ly6C^LO^ memory Tfh (mTfh) cells (Fig 4F). Of note, DAC treated mTh1 and mTfh cells had lower Tbet MFIs (Fig. 4G) which has been linked to Th1 plasticity^40^, suggesting that DAC may decrease Th1 lineage commitment. Overall, these data demonstrate that early DAC treatment during the primary response enhanced memory Tfh cell formation in the spleen.

One possible reason for the increase in memory is that DAC may enhance T cell survival. In fact, our methylation analysis identified *de novo* DMRs at memory-associated genes including *Bcl2*. At 7dpi, we found increased expression of the pro-survival molecules CD127 and Bcl2, but not the pro-apoptotic molecule Bim (Fig. 4H–K). Therefore, DAC treatment enhances memory cell formation in part through improving T cell survival.

### Decitabine treatment during T cell priming enhances functional memory T cell recall responses to heterologous infection with influenza.

**Figure 5.**
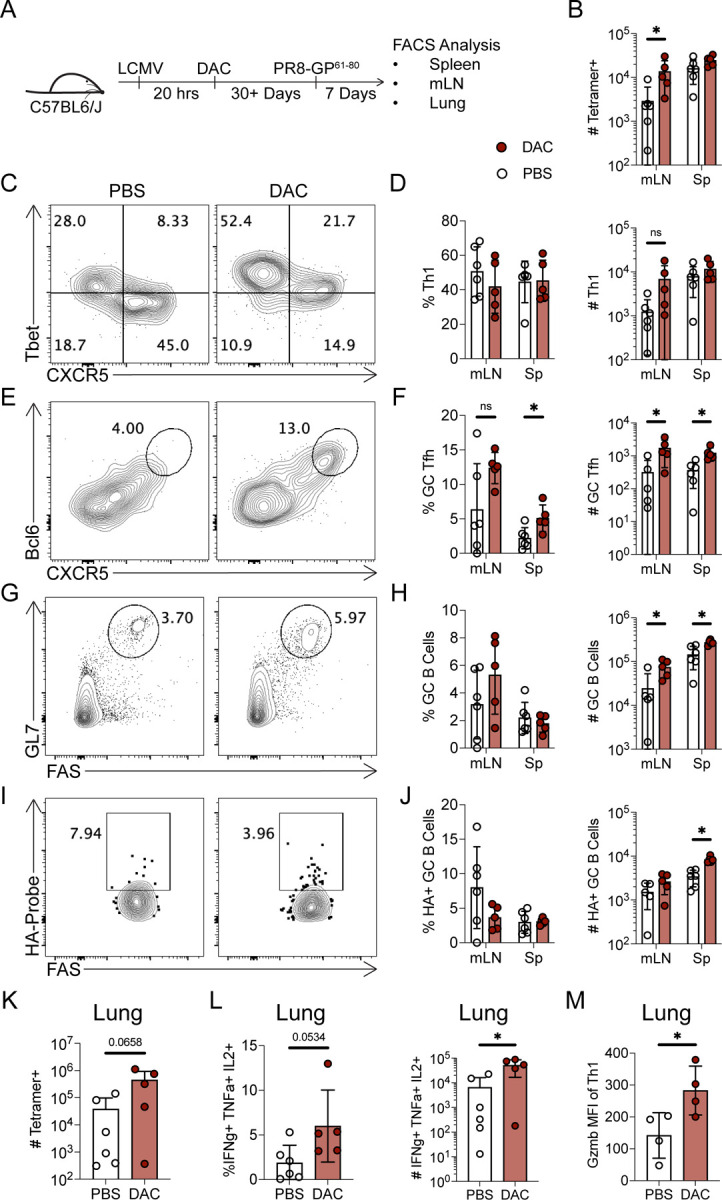
Decitabine treatment during T cell priming enhances functional memory T cell recall responses to heterologous infection with influenza. (A-M) DAC or PBS treated LCMV memory mice were intranasally infected with PR8-GP^61–80^. Spleen (SP), mediastinal lymph node (mLNs), and lung tissues were analyzed 7 days post infection by tetramer staining (I-A(b)-GP^66−77^). (**A**) Experimental schematic. (**B**) Number of tetramer positive CD4+ T cells. (**C**) FACS plots of Tbet and CXCR5, gated on flu specific CD4+ T cells. (**D**) Percent and number of CXCR5− Tbet^HI^ Th1 cells. (E) FACS plots of Bcl6 and CXCR5, gated on flu specific CD4+ T cells. (**F**) Percent and number of CXCR5+ Bcl6^HI^ GC Tfh cells. (**G**) FACS plots of GL7 and Fas, gated on B cells. (**H**) Percent and number of GC B cells. (**I**) FACS plots of HA-probe and FAS, gated on GC B cells. (**J**) Percent and number of HA+ GC B cells. (**K**) Number of tetramer positive cells in the lung. (**L**) Percent and number of polyfunctional cells in the lung. (**M**) Granzyme B MFI of CXCR5− Th1 cells in the lung. All data is gated on antigen specific (I-A(b)-GP^66−77^) CD4+ T cells. Data are representative of two independent experiments. * p<0.05, **p<0.01. Statistical significance was determined by a student’s t test.

Based on the previous results, we reasoned that DAC treatment will result in memory cells that are intrinsically programmed for increased GC Tfh cell generation. To test this idea, SMARTA memory CD4+ T cells were generated that were treated with either DAC or PBS at 20 hpi. At 30 dpi, mice were intranasally infected with a recombinant strain of influenza PR8 that contains the GP^61−80^ epitope^41^. We analyzed spleen, mLNs, and lung tissues at 7 dpi (Fig 5A). DAC treated recipients had more secondary (2°) effector SMARTA cells in the mLNs but not in the spleen (Fig. 5B). While there was no difference in the number of 2° Th1 cells in the mLNs of DAC treated mice (Fig. 5C–D), there was an increase in the percent and number of 2° Bcl6^HI^ GC Tfh cells found in the mLNs and spleen of DAC treated mice (Fig. 5E–F). Supporting this observation, we found a greater number of GC B cells in the mLNs and spleen (Fig. 5G–H), and an increase in hemagglutinin-specific GC B cell numbers in the spleens of DAC treated recipients (Fig. 5I–J). Together, these data indicate that early DAC treatment during T cell priming results in memory cells with an intrinsic bias towards GC Tfh cell differentiation that enhances GC B cell responses to influenza.

We also analyzed the lung where we found a trending increase in the number of antigen-specific T cells (p=0.0658) and a significant increase in the number of polyfunctional T cells (Fig. 5K–L). In addition, we observed a significant increase in the granzyme B MFI of Th1 cells in the lung (Fig. 5M). These data suggest that in addition to enhancing the GC Tfh cell response in the mLN, DAC enhanced the Th1 cell response in the lung during a secondary response to viral infection.

### Decitabine treatment during T cell priming impairs the lineage commitment of memory Th1 cells

**Figure 6.**
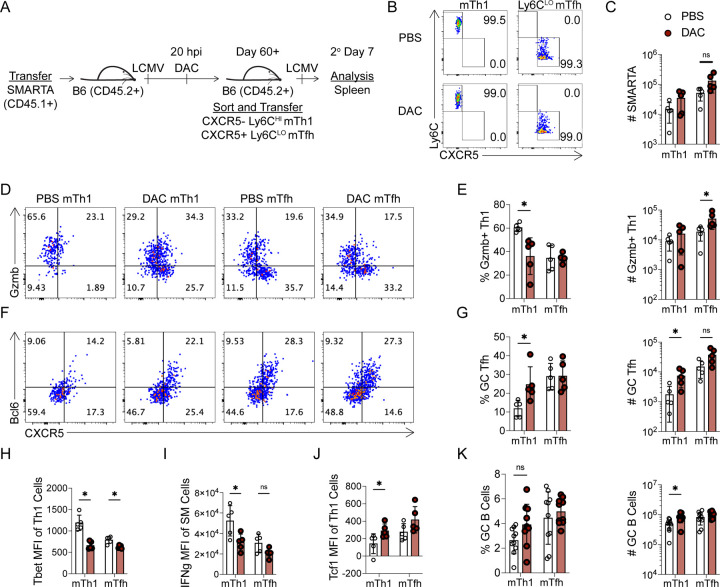
Decitabine treatment during T cell priming impairs the lineage commitment of memory Th1 cells. (A-H) DAC or PBS treated SMARTA memory cells were enriched and sorted to greater than 98% purity. Populations were independently transferred into naïve B6 mice which were subsequently infected with LCMV. SMARTA T cell memory recall responses were analyzed 7 dpi. (**A**) Experimental schematic. (**B**) FACS sort purity analysis. (**C**) Number of SMARTA cells in the spleens. (**D**) Representative FACS plots of Granzyme B (Gzmb) and CXCR5 staining for Th1 identification, gated on SMARTA T cells. (**E**) Percent and Number of CXCR5− Gzmb+ Th1 cells. (**F**) FACS plots of Bcl6 and CXCR5 for GC Tfh identification, gated on SMARTA T cells. (**G**) Percent and number of CXCR5+ Bcl6^HI^ GC Tfh cells. (**H**) Tbet MFI of CXCR5− Th1 SMARTA cells. (**I**) IFNγ MFI of IFNγ+ SMARTA cells. (**J**) Tcf1 MFI of CXCR5− Th1 SMARTA cells. (**K**) Percent and number of GC B cells. For K, data are pooled from two independent experiments. Data are representative of two independent experiments. * p<0.05, **p<0.01. Statistical significance was determined by a student’s t test.

Several pieces of data from the DAC model suggests that it could be interfering with memory CD4+ T cell lineage commitment. First, we observed reductions in Th1 transcription factors including Blimp1 (Fig. S1M) and Tbet (Fig. 4G). In addition, we observed enhanced memory Tfh cell formation (Fig. 4F) and increased GC Tfh cell differentiation during the memory recall response (Fig 5F). Therefore, we next asked whether early DAC treatment during priming can alter the lineage commitment of mTh1 and mTfh cells. SMARTA memory T cells (Day 60+), previously generated in early DAC or PBS treated mice, were sorted into CXCR5+ Ly6C^LO^ mTfh and CXCR5− Ly6C^HI^ mTh1 populations at >98% purity, independently transferred into naïve B6 mice, and infected with LCMV (Fig. 6A–B). We analyzed the recall response at 7dpi. DAC treatment during priming did not significantly alter 2° SMARTA accumulation (Fig. 6C). In agreement with previous literature, we found that mTh1 cells (PBS treated) were lineage committed^4, 5^. The majority of mTh1 progeny cells upregulated granzyme B and took on a Th1-like phenotype (Fig. 6D–E). Additionally, mTfh cells (PBS treated) produced the most 2° GC Tfh cells (Fig. 6F–G). We then analyzed the effects of DAC treatment on the mTh1 recall response. As a percentage of SMARTA cells, DAC treated mTh1 cells produced significantly fewer granzyme B+ CXCR5− Th1 cells (Fig 6D–E). This correlated with reduced expression of Tbet and IFNγ and increased expression of Tcf1 in 2° Th1 cells (Fig. S2H–J). Together, these data suggest impaired 2° Th1 responses in recipients of DAC treated mTh1 cells. We next analyzed the lineage commitment of mTh1 cells after DAC treatment. DAC treated mTh1 cells produced significantly more 2° GC Tfh cells by both percent and number (Fig. 6F–G). Additionally, DAC treatment enhanced the ability of mTh1 cells to support GC B cell responses. There was a trending increase in the percent of GC B cells from DAC treated mTh1 recipients (p=0.055), and a significant increase by number (Fig. 6K). Overall, these data demonstrate that early DAC treatment during T cell priming alters the lineage commitment and function of mTh1 progeny cells during the memory recall response.

In comparison to the mTh1 cells, DAC treatment had a less remarkable effect on mTfh cell function and lineage commitment. DAC treated mTfh cells produced a similar frequency and number of GC Tfh cells (Fig. 6F–G). There was an increase in the number of 2° Th1 cells in recipients of DAC treated mTfh cells (Fig. 6D–E); However, 2° Th1 cells from DAC treated mTfh cells expressed less Tbet (Fig. 6H). Thus, DAC treatment early during priming had only a minor impact on the phenotype and function of mTfh progeny cells during the memory recall response.

### Dnmt3a restricts GC Tfh cell differentiation in a cell-intrinsic manner

**Figure 7.**
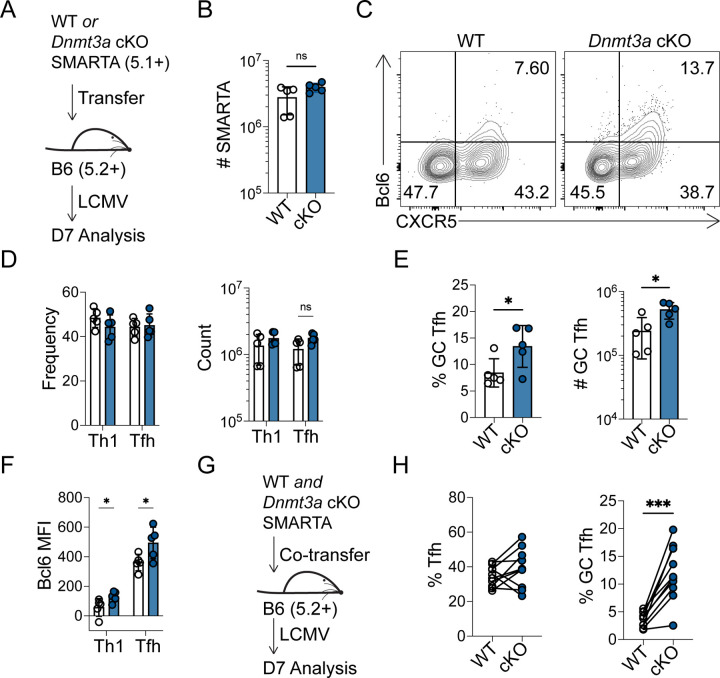
Dnmt3a restricts GC Tfh cell differentiation in a cell-intrinsic manner. (A-F) WT or KO SMARTA mice were treated with tamoxifen to induce Dnmt3a deletion then transferred into B6 mice. Mice were infected with LCMV and CD4+ T cell responses were analyzed 7 dpi. (**A**) Experimental schematic. (**B**) Number of SMARTA T cells. (**C**) Representative FACS plots of Bcl6 and CXCR5, gated on SMARTA T cells. (**D**) Percent and number of CXCR5+ Tfh or CXCR5− Th1 SMARTA cells. (**E**) Percent and number of CXCR5+ Bcl6^HI^ GC Tfh SMARTA cells. (**F**) Bcl6 MFI of Tfh and Th1 SMARTA cells. (G-H) WT or KO SMARTA mice were treated with tamoxifen to induce *Dnmt3a* deletion. Mice were mixed at a 1 to 1 ratio and subsequently transferred into B6 mice. Mice were infected with LCMV and CD4+ T cell responses were analyzed 7 dpi. (**G**) Experimental schematic. (**H**) percent CXCR5+ Tfh and CXCR5+ Bcl6^HI^ GC Tfh SMARTA cells. Data are representative of two independent experiments. * p<0.05, **p<0.01. For A-F, statistical significance was determined by a student’s t test. For G-H, statistical significance was determined by a paired Student’s t test.

Thus far, our data demonstrates loss of memory Th1 cell lineage commitment following early DAC treatment (Fig. 6), which may be linked to inhibition of *de novo* methylation (Fig. 1). Previous studies demonstrate that Dnmt3a is the predominantly upregulated *de novo* methyltransferase in activated CD4+ T cells^25, 26^, therefore, we next sought to determine the effects of *Dnmt3a* deletion on T helper differentiation and lineage commitment in memory cells following viral infection. We bred mice to generate a mouse line containing the SMARTA TCR, *ERT2-Cre*, and *Dnmt3a flox/flox* alleles. Donor SMARTA *ERT2-Cre* mice (either *Dnmt3a*+/+ or *flox/flox*) were treated with tamoxifen to induce Cre-mediated recombination. Naïve SMARTA cells from either WT or *Dnmt3a* conditional knockout (cKO) donor mice were adoptively transferred into B6 recipients that were then infected with LCMV (Fig. 7A). At 7 dpi, loss of Dnmt3a protein expression was validated by flow cytometry (Fig. S2A). In addition, we verified recombination-mediated removal of the LoxP-flanked *Dnmt3a* locus in cKO cells by qPCR (Fig. S2B). We found no difference in SMARTA accumulation (Fig. 7B) or in Tfh/Th1 differentiation in the spleen (Fig. 7C–D). There was also no difference in the number of polyfunctional cells (Fig. S2C). However, we found a significant increase in the percent and number of Bcl6^HI^ GC Tfh cells (Fig. 7C and 7E). In agreement with this, we found that cKO Tfh and Th1 cells expressed significantly more Bcl6 compared to WT cells (Fig. 7F). Next, we co-transferred equal numbers of WT and cKO SMARTA cells (with distinct congenic markers) into B6 mice and infected with LCMV (Fig. 7G). While there was no difference in the percent of Tfh cells, *Dnmt3a* cKO SMARTA cells exhibited a significant increase in the percent of GC Tfh cells at 7 dpi (Fig. 7H). These experiments demonstrate that Dnmt3a-dependent intrinsic programing restricts GC Tfh differentiation during viral infection.

### Dnmt3a is dispensable for memory cell formation

We next analyzed the effect of *Dnmt3a* deficiency on memory formation. In initial adoptive transfer experiments, which were carried out using B6 mice as recipients, WT and *Dnmt3a* cKO SMARTA cells were detectable at effector timepoints but undetectable at memory timepoints (Figure S2D–E). This issue was resolved by transferring WT and cKO SMARTA cells into Cre+ B6 recipients (Fig. S2D–E). Thus, we transferred either WT or cKO SMARTA cells into Cre+ B6 recipients, infected with LCMV, and analyzed the memory response in the spleen at 60+ dpi (Fig. S2F). Loss of *Dnmt3a* had no effect on the number of SMARTA memory cells (Fig. S2G). Phenotypically, we observed an increase in the frequency but not count of CXCR5− Ly6C^LO^ cells (Fig. S2H). In addition, cKO Ly6C^HI^ mTfh and Ly6C^LO^ mTfh cells expressed less Tcf1 (Fig. S2I), while cKO mTh1 cells expressed less Tbet, Runx2, and Runx3 (Fig. S2J). Therefore, while *Dnmt3a* loss did not alter memory formation, it did reduce expression of Tfh and Th1 transcirption factors at memory timepoints, suggesting impaired lineage commitment of *Dnmt3a* cKO mTfh and mTh1 cells.

### Dnmt3a balances the plasticity and functionality of memory Tfh and Th1 cells

**Figure 8.**
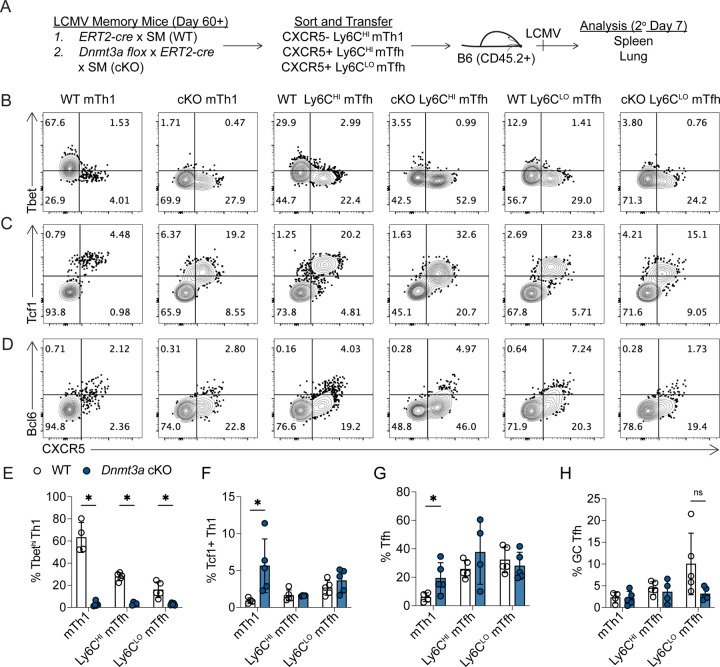
Dnmt3a balances the plasticity and functionality of memory Tfh and Th1 cells. (A-H) WT or KO memory SMARTA cells were generated following LCMV infection. At 60+ dpi, spleens were extracted and SMARTA cells were sorted into CXCR5− Ly6C^HI^ mTh1, CXCR5+ Ly6C^HI^ mTfh, and CXCR5+ Ly6C^LO^ mTfh, independently transferred into naïve recipients, and mice were infected with LCMV. The recall analysis was analyzed in the spleen and lung at 7 dpi. (**A**) Experimental schematic. Representative FACS plots of (**B**) Tbet by CXCR5, (**C**) Tcf1 by CXCR5, and (**D**) Bcl6 by CXCR5. All FACS plots are gated on SMARTA cells. Quantification of the percent (**E**) CXCR5− Tbet^HI^ Th1 cells, (**F**) Tcf1+ Th1 cells, (**G**) CXCR5+ Tfh cells, and (**H**) CXCR5+ Bcl6^HI^ GC Tfh cells. Data are representative of three independent experiments. * p<0.05, **p<0.01. Statistical significance was determined by a student’s t test.

To address the effects of *Dnmt3a* deletion on lineage commitment of memory T cells, WT or *Dnmt3a* cKO SMARTA memory cells were FACS sorted into mTh1, Ly6C^HI^ mTfh and Ly6C^LO^ mTfh subsets (Fig. S3A), and transferred into B6 recipients that were then infected with LCMV. The spleen and lung tissues were harvested at 7 dpi (Fig. 8A). Across three repeat experiments, we observed an inconsistent reduction in the number of cKO SMARTA cells in the spleen (Fig. S3B). Next, we analyzed the effect of *Dnmt3a* deletion on the mTh1 recall response. *Dnmt3a* cKO mTh1 cells produced significantly fewer Tbet^HI^ Th1 2° effector cells (Fig. 8B, E), and more Tcf1+ Th1 cells (Fig. 8C, F) compared to WT cells. This correlated with reduced expression of Tbet, IFNγ, and granzyme B in the spleen (Fig. S3C–E) as well as reduced expression of granzyme B in the lung (Fig. S3E). We next analyzed the plasticity of cKO mTh1 cells and found a significant increase in CXCR5+ Tfh cells (Fig. 8D, G). Surprisingly however, despite this increase in 2° CXCR5+ Tfh cells there was no increase in 2° Bcl6^HI^ GC Tfh cells generated from cKO mTh1 cells (Fig. 8D, H), and 2° Tfh cells from cells expressed less Bcl6 (Fig. S3F). These data demonstrate that Dnmt3a restrains the plasticity and enhances the functionality of mTh1 cells, suggesting that Dnmt3a is required to silence the Tfh transcriptional program in mTh1 cells so that they can recall their specialized effector functions during the memory recall response.

We then analyzed the effect of *Dnmt3a* deletion on the mTfh recall response. While cKO Ly6C^LO^ mTfh produced similar frequencies of 2° CXCR5+ Tfh cells (Fig. 8G), there was a trending reduction in 2° Bcl6^HI^ GC Tfh cells (p=0.065) (Fig. 8D, H), and 2° Tfh cells expressed significantly less Bcl6 and Tcf1 (Fig. S3F–G). This impairment in the functional Tfh response from cKO mTfh subsets was not due to enhanced plasticity. To the contrary, cKO Ly6C^LO^ mTfh and Ly6C^HI^ mTfh cells also produced significantly fewer 2° Tbet^HI^ Th1 cells (Fig. 8B, E). Together, these findings indicate that Dnmt3a is essential for mTfh functionality while having little effect on mTfh plasticity, suggesting that Dnmt3a is required to silence repressors of *Bcl6* to enable GC Tfh differentiation during the memory recall response.

We used an additional Cre model system (SMARTA x *CD4-Cre* x *Dnmt3a flox/flox*) to validate our results. We FACS sorted SMARTA memory cells that were either WT and *Dnmt3a* (*CD4-Cre*) KO, independently transferred them into B6 recipients, and infected with LCMV (Fig. S3H). We observed that 2° *Dnmt3a* (*CD4-Cre)* KO SMARTA cells had trending reductions in GC Tfh and significant reductions in Bcl6 expression for Tfh cells (Fig. S3I–J). Importantly, we found a significant reduction by percent and a trending reduction by number (p=0.075) of GC B cells (Fig. S3K). We also found evidence of impaired Th1 cell responses (Fig. S3L–N). Therefore, we concluded that *Dnmt3a* (*CD4-Cre*) KO memory SMARTA cells were worse at supporting GC B cell responses.

### Dnmt3a silences genes associated with alternative T helper lineages in Tfh and Th1 cells

**Figure 9.**
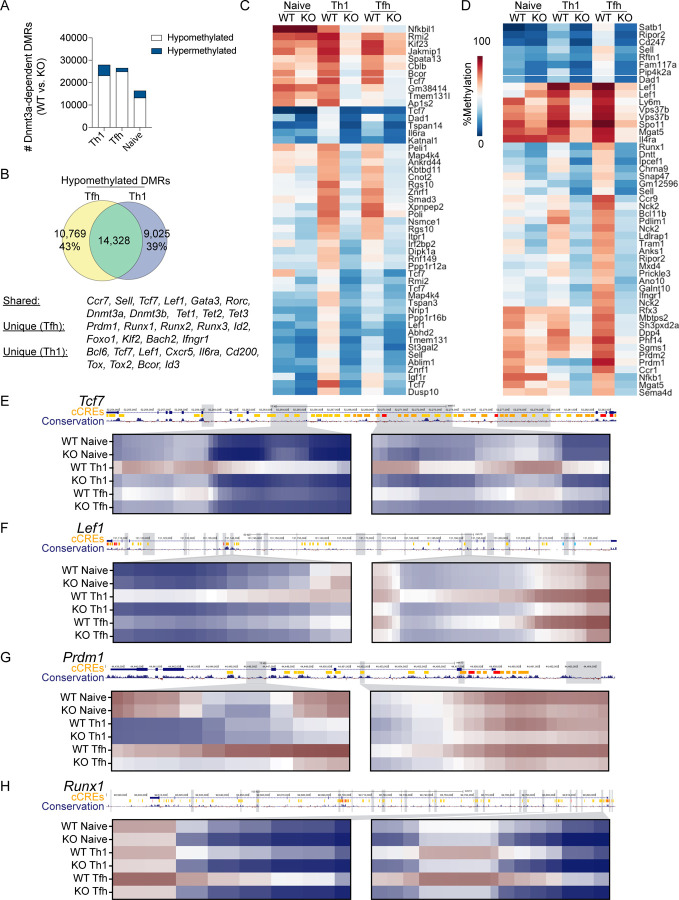
Dnmt3a silences genes associated with alternative T helper lineages in Tfh and Th1 cells. (A-H) Whole genome enzymatic methylation sequencing was performed on WT and *Dnmt3a* cKO CXCR5+ Tfh and CXCR5− Th1 cells sorted at 7 days post LCMV infection. WT and cKO naïve CD4+ T cells were also analyzed as controls. Differentially methylated regions (DMRs) were called between WT and cKO cells of the same lineage. (**A**) Quantification of the total number of DMRs per cell type (WT vs cKO). Hypermethylated regions are blue and hypomethylated regions are white. (**B**) Venn diagram of overlapping hypomethylated DMRs in Tfh and Th1 cells. Heatmaps of (**C**) the top 50 Th1-specific DMRs and (**D**) the top 50 Tfh-specific DMRs. DMRs were ranked by mean difference between WT Tfh and WT Th1 cells. UCSC genome browser snapshots of (**E**) *Tcf7*, (**F**) *Lef1*, (**G**) *Prdm1*, and (**H**) *Runx1*. We also show tracks for ENCODE identified cis-candidate regulatory elements (cCREs) and mammalian conservation. Grey boxed identify hypomethylated DMRs. In the heatmaps below, select DMRs are shown. Each column represents a CpG site across the DMR.

*Dnmt3a* deficiency impaired the lineage commitment and functionality of mTh1 cells, which we hypothesized was due to loss of *de novo* methylation programing at genes associated with the Tfh transcriptional program. In addition, *Dnmt3a* deficiency impaired the functionality of mTfh cells, and we hypothesized that this was due to loss of *de novo* methylation programing at genes encoding *Bcl6* repressors. To determine whether Dnmt3a was necessary for *de novo* methylation at genes associated with alternative T helper lineages, we performed whole genome enzymatic methylation sequencing (WGEM-seq) on WT and *Dnmt3a* (*CD4-Cre*) KO Tfh and Th1 cells sorted from LCMV infected mice at 7 dpi. DMRs were identified between WT and KO cells of the same lineage. This analysis revealed 26,513 DMRs in Tfh cells (WT vs KO) and 27,937 DMRs in Th1 cells (WT vs KO) (Fig 9A). The majority of DMRs were hypomethylated in KO compared to WT and occurred within intragenic regions (Fig. 9A, S4A). Dnmt3a-dependent DMRs were associated with enrichment of H3K4me1 peaks as well as ETS and RUNX motifs, which is consistent with T cell regulatory regions (Fig. S4B–C)^42, 43^. Importantly, most *de novo* DMRs were lost in KO Tfh and Th1 cells compared to their WT counterparts (Fig. S4D). These data indicate that Dnmt3a is the predominant *de novo* methyltransferase in Tfh and Th1 cells and that it is involved in silencing T cell specific regulatory regions.

Analysis of *de novo* methylated regions demonstrated extensive programing that was distinct to Tfh and Th1 cells (Fig. 1B–D). Therefore, we next examined whether Dnmt3a-dependent programing was unique between Tfh and Th1 cells. In agreement with our earlier analysis, we found that the majority of Th1 and Tfh DMRs were shared (Fig. 9B). This included DMRs at genes associated with naïve and memory cells (*Ccr7, Sell, Tcf7, Lef1, Id3*), Th2 and Th17 differentiation (*Gata3, Rorc*), and DNA methylation programming (*Dnmt3a, Dnmt3b, Tet2*). We also found extensive lineage-specific methylation (Fig. 9B–D). Heatmap analysis of the top 50 Th1-specific DMRs included several genes which are involved in Tfh differentiation, including *Tcf*7, *Lef1, Il6ra* and *Bcor* (Fig. 9C). We further analyzed select DMRs at the *Tcf7, Lef1* and *Bcl6* loci, revealing extensive *de novo* methylation at intronic regions with high-degrees of mammalian conservation (Fig. 9E–F, S4E). To our surprise, the first intron of *Bcl6* was more methylated in Tfh cells (Fig. S4E). Additionally, motif analysis found specific enrichment of Tcf1 motifs within 24% of Dnmt3a DMRs in Th1 cells (Fig. S4C). In Tfh cells, heatmap analysis of the top 50 DMRs included several genes which are involved in Th1 differentiation, including *Prdm1, Runx1*, and *Ifngr1* (Fig. 9D). We chose to further analyze the genes *Prdm1, Runx1*, and *Runx2*, revealing extensive *de novo* methylation that occurred at intronic regions with a high degree of mammalian conservation (Fig. 9G–H, S4F). Motif analysis found specific enrichment of Runx1 motifs within 23% of Dnmt3a DMRs in Tfh cells (Fig. S4C). Together, these data suggest Dnmt3a is important for silencing genes associated with alternative lineages.

To determine genes where Dnmt3a may silence expression via *de novo* methylation in primary effector cells, we performed RNA sequencing on Tfh and Th1 cells sorted from LCMV infected mice at 7 dpi. Differentially expressed genes (DEGs) were identified between WT and *Dnmt3a* KO cells of the same cell type. We found 1126 Tfh (WT vs KO) and 58 Th1 DEGs (WT vs KO) (Fig. S4G). We identified genes that were upregulated and hypomethylated in cKO Th1 cells. This included the Tfh associated gene *Lef1*, which was more expressed by transcript and protein levels (Fig. S4H–I). *Dnmt3a* KO Tfh cells upregulated and hypomethylated several Th1 associated genes, including *Runx1* which was more highly expressed by transcript and protein levels compared to WT cells (Fig. S4J–K). Thus, Dnmt3a-dependent methylation silences genes associated with alternative T helper lineages in effector Tfh and Th1 cells.

These observations also raised the possibility that *Dnmt3a* KO Tfh cells may fail to silence genes that counteract Tfh programing, and that this may counteract *Bcl6* upregulation and GC Tfh differentiation during the memory recall response in *Dnmt3a* KO cells (Fig. 8). Other more direct mechanisms may play a role in regulating *Bcl6* re-expression. For instance, we found evidence for Dnmt3a-dependent intronic methylation at the *Bcl6* locus in Tfh cells (Fig. S4E). One paper reported a similar finding when comparing human GC B cell and plasma cell lines, and the authors argue that CTCF-mediated repression of *Bcl6* is impeded by intronic methylation^44^. Therefore, we first asked whether our intronic DMRs overlapped with the human CpG islands interrogated in their study using LiftOver. In fact, we found that three of the assayed human CpG islands overlapped with two Dnmt3a-dependent DMRs identified in our study (Fig. S4E). In addition, we identified several CTCF motifs within our intronic *Bcl6* DMRs in Tfh cells. Next, using publicly available CTCF ChIP-seq data (GSM3498276) from murine Naïve CD4+ T cells^45^, we found evidence of two CTCF peaks overlapping with two Dnmt3a-dependent DMRs (Fig. S4E). Importantly, this region is unmethylated in naïve CD4+ T cells (Fig. S4E), strongly suggesting that this mechanism is conserved. Therefore, the impairment in Bcl6 upregulation in *Dnmt3a* deficient 2° Tfh cells (Fig. 6) may not only be due to failure to silence repressors of *Bcl6*, but also due to lack of methylation at a *Bcl6* silencer region.

### Early decitabine treatment partially inhibits Dnmt3a-mediated methylation programming at Tfh-associated genes

**Figure 10.**
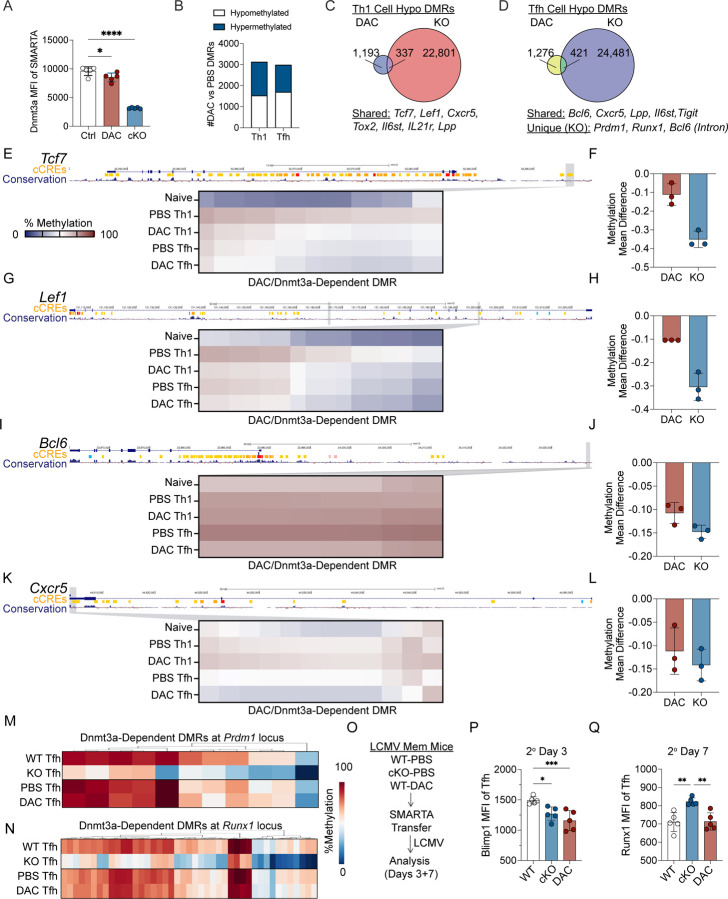
Early decitabine treatment partially inhibits Dnmt3a-mediated methylation programing at Tfh-associated genes. (**A**) WT and *Dnmt3a* cKO SMARTA cells were adoptively transferred into B6 recipients and intravenously infected with LCMV. WT cells received either PBS or DAC and cKO cells received PBS. At 3dpi, we analyzed the T cell response in the spleen. The Dnmt3a MFI of WT, cKO or DAC treated SMARTA T cells is shown in the figure. (B-I) Whole genome enzymatic methylation sequencing was performed on PBS or DAC treated Tfh and Th1 cells sorted at 7 days post LCMV infection. Differentially methylated regions (DMRs) were called between PBS and DAC treated cells of the same lineage. (**B**) Quantification of the number and directionality of DMRs. (C-D) Overlap analysis was performed on DAC-dependent hypomethylated DMRs and Dnmt3a-dependent hypomethylated DMRs identified from (**C**) Th1 or (**D**) Tfh cells. (E, G, I and K) UCSC genome browser snapshots of select DMRs. All shown DMRs are DAC-dependent DMRs (PBS vs DAC) that overlap with Dnmt3a-dependent DMRs (WT vs KO). (F, H, J, and L) the mean difference in methylation (KO – WT or DAC – PBS) are shown across the select DAC-dependent DMR. (**E**) DAC dependent DMR in Th1 cells at *Tcf7*, and (**F**) quantification of mean difference across the *Tcf7* DMR. (**G**) DAC dependent DMR in Th1 cells at *Lef1*, and (**H**) quantification of mean difference across the *Lef1* DMR. (**I**) DAC dependent DMR in Tfh cells at *Bcl6*, and (**J**) quantification of mean difference across the *Bcl6* DMR. (**K**) DAC dependent DMR in Tfh cells at *Cxcr5*, and (**L**) quantification of mean difference across the *Cxcr5* DMR. (M and N) Heatmaps show mean methylation for all Dnmt3a-dependent DMRs associated with (**M**) *Prdm1* and (**N**) *Runx1*. (O-Q) LCMV memory T cells (WT, cKO or DAC) were generated, CD4+ T cells were enriched, adoptively transferred into naïve B6 mice and infected with LCMV. (**O**) Experimental schematic. MFI analysis of Tfh cells for (**P**) Blimp1 and (**Q**) Runx1. For all locus specific heatmaps, we also show tracks for ENCODE identified cis-candidate regulatory elements (cCREs) and mammalian conservation. Grey boxed identify DMRs, which are highlighted below. Each column represents a CpG site across the region. For A, data are representative of a single independent experiment. * p<0.05, **p<0.01, ***p<0.005, **** p<0.0001. Statistical significance was determined by a one-way ANOVA.

We next determined whether DAC treatment inhibited Dnmt3a. DAC is a cytidine analog that is incorporated into DNA during replication, resulting in the formation of DAC-Dnmt3a adducts that are excised and degraded^29–31^. To address whether Dnmt3a was inhibited in DAC treated SMARTA cells, we set up an experiment to measure Dnmt3a levels within early activated T cells. Naïve SMARTA cells were transferred into B6 mice, infected with LCMV, and treated with DAC at 20 hpi. At 3 dpi, we measured Dnmt3a protein levels within activated SMARTA cells. We found a small but significant decrease in the MFI of Dnmt3a within DAC treated SMARTA cells (Fig. 10A). These data suggest that DAC treatment led to Dnmt3a degradation within SMARTA T cells, although inhibition was likely temporary and incomplete.

We therefore decided to determine if DAC treatment impaired Dnmt3a-mediated methylation programing. To determine the effect of DAC treatment on DNA methylation within SMARTA cells, we performed WGEM-seq on DAC or PBS treated Tfh and Th1 cells at 7 dpi. This analysis revealed 2984 Tfh DMRs (PBS vs DAC) and 3127 Th1 DMRs (PBS vs DAC) that were DAC-dependent (Fig. 10B). Surprisingly, approximately half of the DMRs were hypermethylated (Fig. 10B), suggesting that DAC treatment may have indirect effects. To determine if DAC-dependent DMRs were shared with Dnmt3a-dependent DMRs, which would suggest Dnmt3a inhibition, we next identified overlapping hypomethylated DMRs. This analysis identified 421 Tfh DMRs and 337 Th1 DMRs that were hypomethylated in both the DAC treatment and *Dnmt3a* KO models (Fig. 10C–D). Overall, these data support the conclusion that DAC treatment inhibited Dnmt3a-mediated programing in differentiating Tfh and Th1 cells, but to a lesser degree compared to the *Dnmt3a* KO model.

We next analyzed Th1 cells to determine whether DAC treatment inhibited Dnmt3a-mediated silencing of Tfh genes. In Th1 cells, DAC treatment and *Dnmt3a* deficiency both lead to hypomethylation of Tfh genes, including *Tcf7, Lef1, Cxcr5, Tox2, and Il6st* (Fig. 10C). Specifically, DAC partially inhibited Dnmt3a-dependent *de novo* methylation at a region upstream of *Tcf7* that overlapped with a candidate cis regulatory element (cCRE) (Fig. 10E–F). DAC treatment also partially inhibited Dnmt3a-dependent *de novo* methylation at two intronic regions in *Lef1*, including one that overlapped with a cCRE (Fig. 10G–H). Of note, DAC treated and *Dnmt3a* cKO mTh1 cells upregulated Tcf1 (Fig. 6J; 8F). Thus, decitabine treatment early during priming partially inhibited Dnmt3a-dependent methylation at Tfh-associated genes, including *Tcf7* and *Lef1*, impairing memory Th1 cell lineage commitment.

Analysis of the 421 Tfh DMRs that were hypomethylated and shared in both DAC and *Dnmt3a* cKO models revealed that several Tfh genes were hypomethylated, including *Bcl6, Cxcr5, Lpp* and *Il6st* (Fig. 10D). At the *Bcl6* locus, we specifically found one upstream region where DAC treatment partially inhibited Dnmt3a-mediated *de novo* methylation (Fig. 10I–J). DAC treatment also partially inhibited Dnmt3a-mediated *de novo* methylation at a region within *Cxcr5* (Fig. 10K–L). Thus, decitabine treatment early during priming partially inhibited Dnmt3a-dependent methylation at Tfh-associated genes, including *Bcl6* and *Cxcr5*, enhancing Bcl6 expression and GC Tfh cell differentiation in Tfh cells.

Our data strongly suggest that DAC treatment impaired *de novo* methylation at only a subset of Dnmt3a-dependent DMRs. Of note, *Dnmt3a* cKO memory T cells fail to upregulate Bcl6 and differentiate into GC Tfh cell (Fig. 8), while DAC treated memory T cells have enhanced Bcl6 expression and GC Tfh cell differentiation (Fig. 5, 6). One possible explanation for this difference is that *Dnmt3a* deletion results in memory Tfh cells that aberrantly upregulate repressors of *Bcl6* and Tfh differentiation, while DAC treated memory Tfh cells properly silence repressors of *Bcl6*. We therefore decided to analyze known *Bcl6* repressors that were targets of Dnmt3a-mediated methylation. Importantly, DAC treatment did not inhibit Dnmt3a-mediated methylation at *Prdm1* (11 DMRs) (Fig. 10M, S5A–B) or *Runx1* (43 DMRs) (Fig. 10N, S5C–D). DAC treatment also did not impair *de novo* methylation at *Foxo1* (26 DMRs), *Runx2* (29 DMRs), *Runx3* (20 DMRs), and *Id2* (3 DMRs) (Fig. S5G). Another possibility is that DAC treatment did not inhibit Dnmt3a-mediated *de novo* methylation within first intron of *Bcl6*. This region inhibits CTCF binding and repression of *Bcl6* and is associated with high expression of *Bcl6* in B cells^44^. In fact, DAC treatment had insignificant effects on the acquisition of Dnmt3a-dependent methylation in the first intron of *Bcl6* (Fig. S5E–F). Combined, these data strongly demonstrate that *Dnmt3a* deficient Tfh cells fail to acquire *de novo* methylation at several genes encoding repressors of Tfh differentiation or function, including *Prdm1, Runx1, Runx2, Runx3, Id2*, and *Foxo1*, as well as a known *Bcl6* silencer region. However, DAC treatment during T cell priming did not inhibit Dnmt3a-mediated *de novo* methylation at repressors of Tfh differentiation, while inhibiting Dnmt3a-mediated methylation at other Tfh-associated genes, explaining the difference in GC Tfh differentiation during the memory recall response.

We next determined if *Dnmt3a* cKO 2° Tfh cells aberrantly upregulated repressors of *Bcl6*. To address this question, LCMV derived SMARTA memory cells (Day 60) were generated that were either WT (PBS-treated), *Dnmt3a* cKO (PBS-treated), or WT (DAC-treated) early during priming. Memory CD4+ T cells from each group were enriched and transferred into B6 recipients that were subsequently infected with LCMV. We then analyzed the expression of transcription factors that repress Tfh differentiation at days 3 and 7 during the memory recall response (Fig. 10O). Both *Dnmt3a c*KO and DAC treated 2° Tfh cells downregulated Blimp1 (Fig. 10P), as well as Foxo1, Runx2, and Runx3 (Fig. S5H). There was a trending reduction in Id2 expression in both *Dnmt3a* cKO and DAC treated 2° Tfh as well (Fig. S5H). In contrast, *Dnmt3a* cKO 2° Tfh cells aberrantly upregulated Runx1 while DAC treated 2° Tfh cells expressed normal levels of Runx1 relative to the control (Fig. 10Q). These data demonstrate that Dnmt3a is required to silence Runx1 in 2° Tfh cells and suggests that Runx1 may repress *Bcl6* re-expression and GC Tfh cell differentiation during the memory recall response. Furthermore, these findings indicate that DAC treatment derepressed several Tfh genes (*Bcl6* and *Cxcr5*), but did not inhibit *de novo* methylation at genes encoding repressors of Tfh differentiation (*Prdm1* and *Runx1*), that was associated with enhancement of GC Tfh generation during the memory recall response.

## DISCUSSION

Previous literature has demonstrated the existence of memory Tfh and Th1 populations that are intrinsically programmed to recall their specialized effector functions^4–6^. Tet2-dependent demethylation has been implicated in memory Th1 lineage commitment^6^; however, the role of Dnmt3a-mediated *de novo* methylation is unclear. We demonstrate that Dnmt3a mediates lineage-specific *de novo* methylation programing in Tfh and Th1 cells. We argue in favor of a model that Tfh- and Th1-specific *de novo* methylation programing is acquired early during T helper differentiation during the primary response and that such programing is maintained in respective memory Tfh and Th1 cells. In agreement with this point, we find *de novo* methylation at *Dnmt3a* and *Dnmt3b* that silences their expression. We therefore reason that early *de novo* methylation programing during priming may be fundamental to the lineage commitment of memory Tfh and Th1 cells. To this point, we find that Dnmt3a is required to enforce memory Th1 lineage commitment and for highly functional Tfh and Th1 cell responses during the secondary viral challenge. Importantly however, using a methyltransferase inhibitor that only partially inhibited Dnmt3a-mediated methylation programing, we demonstrate enhancement of both mTh1 plasticity and 2° Tfh functionality during the memory recall response. Overall, this study demonstrates that Dnmt3a-mediated methylation programing is a targetable pathway that balances the plasticity and functionality of memory Tfh and Th1 cells.

Our data support a model whereby Dnmt3a-dependent *de novo* methylation silences Tfh-associated genes, including *Tcf7* and *Lef1*, to enforce Th1 cell lineage commitment. In agreement with this, we find that Dnmt3a represses *Lef1* expression during the primary response. During the memory recall response, we find evidence of Dnmt3a-dependent repression of *Tcf7* expression. In addition, our motif analyses reveal that Dnmt3a-dependent Th1 DMRs are enriched for Tcf1/Lef1 motifs. These data strongly implicate Dnmt3a in silencing Tcf1 and Lef1 mediated transcriptional programing in Th1 cells by (1) silencing *Tcf7* and *Lef1* and (2) restraining accessibility to Tcf1/Lef1 binding sites within the genome. Furthermore, we argue that Dnmt3a-mediated repression of the Tfh transcriptional program in memory Th1 cells is required to initiate a highly functional Th1 memory recall response. For instance, we find that Dnmt3a is essential for proper expression of Th1 effector molecules during the memory recall response. Together, these data demonstrate that Dnmt3a restrains the plasticity of memory Th1 cells to enable the functionality of the secondary effector Th1 cells during the memory recall response.

While inhibition of Dnmt3a-mediated methylation programing had less remarkable effects on the lineage commitment of memory Tfh cells, we attribute this to the inherent plasticity of memory Tfh cells relative to memory Th1 cells^4–6^. Manipulation of Dnmt3a-dependent methylation programing was not without consequence, however, and our data strongly implicate Dnmt3a in regulating Tfh functionality. We found that Dnmt3a was essential for GC Tfh differentiation and GC B cell help during the memory recall response. We propose the model that Dnmt3a-mediated *de novo* methylation silences Tfh repressors, including *Prdm1* and *Runx1*. While Dnmt3a is required for *de novo* methylation of *Prdm1*, we did not find evidence that Dnmt3a was required to silence *Prdm1* re-expression in secondary Tfh cells. However, Dnmt3a was required for *de novo* methylating and silencing *Runx1* within secondary Tfh cells. Runx1 motifs were also enriched in Dnmt3a-dependent Tfh DMRs, suggesting that Dnmt3a may counteract Runx1 binding within the genome. The link between Runx1 and repression of the Tfh program is correlative^18, 43, 46, 47^, so further research is needed to determine the specific role of Runx1 in this process. Nevertheless, these data strongly implicate Dnmt3a in silencing Runx1-mediated transcriptional programing in Tfh cells by (1) methylating and silencing *Runx1* and (2) restraining accessibility to Runx1 binding sites within the genome.

In addition, Dnmt3a also directly regulates the *Bcl6* locus in Tfh cells. *Lai et al*. analyzed human GC B cell and plasma cell lines and found an inverse correlation between expression of *Bcl6* and intronic DNA methylation, which they attributed to CTCF binding and repression of *Bcl6* in the absence of intronic DNA methylation^44^. *Pham et al*. demonstrated CTCF binding within the *Bcl6* locus in naïve CD4+ T cells, which we demonstrate occurs within the intronic regions that acquire Dnmt3a-dependent *de novo* methylation in Tfh cells, suggesting that this mechanism may be conserved^45^. Therefore, we argue that Dnmt3a (1) silences repressors of Tfh differentiation, including *Prdm1* and *Runx1*, and (2) inhibits CTCF binding at the *Bcl6* locus, thereby safeguarding the functionality of Tfh cells during the memory recall response.

In our studies, early decitabine treatment during primary viral infection inhibited Dnmt3a-mediated programming in virus-specific CD4+ T cells. However, we cannot rule out that decitabine treatment may also have additional cell-extrinsic effects, or that it inhibited Dnmt1 or Dnmt3b in virus specific CD4+ T cells. Further experimentation is needed to determine the contribution of these other mechanisms to the observed phenotypes following decitabine treatment. In agreement with our preferred model, we observed a reduction in Dnmt3a protein levels in decitabine treated virus-specific CD4+ T cells, and decitabine treatment led to hypomethylation of select regions that acquire Dnmt3a-dependent *de novo* methylation in virus-specific CD4+ T cells. Furthermore, decitabine treatment reproduced several key aspects of the *Dnmt3a* knockout phenotype, including (1) increasing the GC Tfh cell response during the primary infection and (2) enhancing memory Th1 cell plasticity during the memory recall response. Together, these data demonstrate that early decitabine treatment impaired Dnmt3a-mediated methylation programing in virus-specific CD4+ T cells.

Decitabine did not recapitulate all aspects of the *Dnmt3a* cKO phenotype, however. For example, decitabine treatment and *Dnmt3a* deletion had opposing effects on Tfh functionality during the memory recall response. At the methylation level, we found that decitabine treatment only partially inhibited methylation programming at a subset of Dnmt3a-dependent DMRs. For instance, we found that decitabine inhibited Dnmt3a-mediated *de novo* methylation programing at *Tcf7/Lef1* in Th1 cells, and *Bcl6/Cxcr5* in Tfh cells. Importantly, we observed proper Dnmt3a-dependent methylation at *Prdm1* and *Runx1* in decitabine treated Tfh cells, suggesting that they were properly silenced the non-Tfh program. In contrast, deletion of *Dnmt3a* lead to a complete loss of *de novo* methylation at the several Tfh repressors, including *Prdm1, Runx1*, and *Runx2*, as well as aberrant upregulation of Runx1 during the memory recall response. Based on the methylation data, we propose that inhibition of Dnmt3a was temporary and incomplete following decitabine treatment. Therefore, these data strongly suggest that complete inhibition of Dnmt3a-mediated programing via genetic deletion enhances memory T cell plasticity at the expense of a functional memory recall response; however, temporary and partial inhibition of Dnmt3a via decitabine treatment early during the primary response (single dose 20 hours after infecting) enhances both memory T cell plasticity and GC Tfh cell functionality during a secondary recall response (Fig S5I).

Recent studies have demonstrated that increased GC Tfh cell numbers correlate with improved GC B cell responses and the generation of neutralizing antibodies in response to HIV infection or immunization^48–52^. In addition, memory Tfh and B cells interact upon antigen re-encounter and is thought to be of importance in reformation of the germinal center^2^. Targeting Dnmt3a-mediated methylation pathways may be one way to enhance GC Tfh and memory Tfh cell responses and improve humoral immunity to immunizations and infections. Furthermore, targeting DNA methyltransferases may be of benefit to other cell types during the immune response to immunizations and infections. In line with this idea, combined deletion of *Dnmt3a* and *Dnmt3b* in B cells increased GC B cell responses following immunization^53^ and deletion of *Dnmt3a* enhanced memory precursor formation in CD8+ T cells^54, 55^. Our study complements this growing body of literature by demonstrating the potential for pharmacological inhibition of DNA methyltransferases to reprogram T helper responses in favor of GC Tfh and memory Tfh generation. Further research is needed to determine whether targeting Dnmt3a-mediated methylation may constitute a novel strategy for enhancing GC Tfh cell responses and modulate humoral immunity to immunizations and infections.

## MATERIALS AND METHODS

### Mouse Models:

C57BL/6J (CD45.2+) or *R26-CreERT2* mice (CD45.2+) were purchased from The Jackson Laboratory for use as adoptive transfer recipients. For decitabine treatment experiments, SMARTA TCR transgenic mice (CD45.1+) were purchased from The Jackson Laboratory. For Dnmt3a KO experiments, *Dnmt3a*^*fLOx/fLOx*^ mice were either crossed to *R26-CreERT2* mice and SMARTA TCR transgenic mice to generate *Dnmt3a*^*fLOx/fLOx*^ x *R26-CreERT2* x *SMARTA* mice (CD45.1+). *R26-CreERT2* x *SMARTA* mice (CD45.1+; cre+ control mice) and *Dnmt3a*^*fLOx/fLOx*^ x *SMARTA* mice (CD45.1+; flox control mice) were also generated to serve as controls. Tamoxifen (2mg/mouse, dissolved in corn oil) was IP injected into *Dnmt3a*^*fLOx/fLOx*^ x *R26-CreERT2* x *SMARTA* mice (CD45.1+) and control mice for four consecutive days, followed by a 3 day rest period, to induce Dnmt3a deletion. Additionally, *Dnmt3a*^*fLOx/fLOx*^ mice were crossed to *CD4cre* mice and SMARTA TCR transgenic mice to generate *Dnmt3a*^*fLOx/fLOx*^ x *CD4cre* x *SMARTA* mice (CD45.1+). *CD4cre* x *SMARTA* mice (CD45.1+; cre+ control mice) *Dnmt3a*^*fLOx/fLOx*^ x *SMARTA* mice (CD45.1+; flox control mice) were also generated to serve as controls. All animal experiments were conducted in accordance with University of Utah Institutional Animal Care and Use Committee–approved protocols.

### Adoptive Transfer and LCMV Infections:

For all adoptive transfer models, splenocytes were isolated were magnetically enriched using the CD4+ T cell negative selection kit (STEMCELL Technologies). For analysis at 7, 8, or 10 days post primary infection, 2×10^4^ naïve Va2+ CD4+ T cells were transferred into C57BL/6J recipients (CD45.2+). For analyses at memory timepoints, 2×10^5^ naïve Va2+ CD4+ T cells were transferred into C57BL/6J recipients (CD45.2+; decitabine experiments) or *R26-CreERT2* recipients (CD45.2+; Dnmt3a KO experiments). The next day, mice were infected with 2×10^5^ pfu of LCMV Armstrong via an IP injection.

### Influenza Infections:

C57BL/6J mice were purchased from The Jackson Laboratory C57BL/6J and were intranasally infected with 500 TCID_50_ of PR8 (H1N1) strain. For intranasal infections, 138 mice were anesthetized with concurrent administration of aerosolized isoflurane and oxygen using a COMPAC Anesthesia Center (VetEquip). Prior to euthanasia, mice were intravenously injected with 2 μg α-CD45-FITC (30-F11, Tonbo Biosciences) antibody to detect remaining circulating cells in lung samples.

### Decitabine Treatment:

5’-aza-2′-deoxycytidine (Dectabine) was purchased from Sigma Aldrich and was dissolved in sterile PBS prior to intraperitoneal injection 20 hours post infection. For influenza experiments, decitabine was given at 0.375mg/kg (~7.5mg DAC/mouse). For LCMV experiments, decitabine was given at 0.75mg/km (~15mg DAC/mouse).

### Tissue Processing

Single-cell suspensions of pooled mediastinal lymph nodes or pooled inguinal and lumbar lymph nodes were prepared using 70-μm cell strainers. Single-cell suspensions of spleens were prepared using 70-μm cell strainers and red blood cells lysed by incubation in Ammonium-Chloride-Potassium (ACK) Lysing Buffer (Life Technologies). Single-cell suspensions of lungs were prepared by digestion with 0.25mg/ml Collagenase IV and 15 μg/ml DNase for 1 hour at 37°C, then manually homogenized and red blood cells lysed by incubation in ACK Lysing Buffer and then cells were filtered using 70-μm cell strainers. Cell suspensions were resuspended in RPMI 1640 media supplemented with 5% fetal bovine serum (FBS) prior to FACS staining.

### Flow Cytometry and Cell Sorting:

Cells were surface stained in PBS supplemented with 2% fetal bovine serum (FBS) for 30 minutes on ice. Antibodies include CD4+ (RM4–5), CD8 (53–6.7), CD44 (IM7), CD45.1(A20), CD45.2(104), Ly6c (HK1.4), CXCR5 (L138D7), PD-1 (29F.1A12),CD25 (PC61),CD127 (SB/119),CD95 (Jo2), GL7 (GL7), PNA (FL-1071), CD19 (eBio1D3 (1D3)), B220 (RA3–6B2), IgD (11–26c.2a), and CD138 (281–2). Cells were subsequently fixed with the Foxp3 Permeabilization/Fixation kit and protocol (eBioscience) for staining intracellular factors. Intracellular antibodies include Dnmt3a (D23G1), Bcl6 (K112–91), Tcf1 (S33–966), Tbet (4B10), Blimp1 (5E7), Bcl2 (BCL/10C4), Bim (C34C5), Foxp3 (FJK-16s), Granzyme B (GB12), Runx1 (RXDMC), Runx2 (D1L7F), Runx3 (R3–5G4), Lef1 (C12A5), and Foxo1 (C29H4). For intracellular cytokine staining, cells were first stimulated with GP_61–80_ peptide and brefeldin A (GolgiPlug, BD Biosciences) for 5 hours. Cells were then stained for surface antigens followed by permeabilization, fixation, and staining using the Cytofix/Cytoperm kit (BD Biosciences). Antibodies included IFNγ (XMG1.2), TNFα (MP6-XT22), and IL-2 (JES6–5H4). For detection of antigen specific CD4+ T cells, tetramer staining was performed prior to surface staining using I-A(b) LCMV GP^66−77^, I-Ab NP^311−325^, or I-A(b) CLIP^87−101^ (NIH Tetramer Core). Cells were incubated at 37 degrees for 1 hour in RPMI supplemented with 10% fetal bovine serum. For influenza HA-specific B cell staining, recombinant HA protein from A/Puerto Rico/8/1934 (H1N1) virus strain (Immune Technology Corp., #IT-003–0010ΔTMp) was biotinylated with 80-fold molar excess of NHS-PEG4-Biotin solution from the EZ-Link NHS-PEG4-Biotin kit (ThermoFisher, #A39259). Excess biotin was removed by buffer exchange of protein into sterile 1X PBS using Zeba Spin Desalting Columns, 7K MWCO (ThermoFisher, #89882). Cells were stained on ice for 30min in FACS buffer with 1:100 dilutions of biotin-conjugated-HA and purified rat anti-mouse CD16/CD32 (Mouse BD Fc Block, Clone 2.4G2, BD Biosciences), then stained on ice for 30min in FACS buffer with 1:1000 dilution of allophycocyanin (APC)-conjugated streptavidin. Flow cytometry data were collected on FACSCanto and LSRFortessa X-20 (BD Bioscience). Flow cytometry data were analyzed using FlowJo software (TreeStar).

### Cell Sorting

For cell sorting at memory timepoints, SMARTA cells from spleens were stained using PE conjugated anti-CD8, anti-CD45.2, and anti-B220 antibodies. Stained cells were then enriched using the Anti-PE MicroBeads kit (Miltenyi Biotec). Cells were subsequently surface stained for FACS sorting. Cell sorting was performed using FACSAria (BD Bioscience).

### RNA isolation and RNA sequencing:

RNA was isolated from sorted CD4+ T cells using an RNA MiniPrep kit (Zymo). RNA-seq libraries were generated using the NEBNext Ultra II Directional RNA Library Kit (New England BioLabs). Prepared libraries were sequenced using an Illumina NovaSeq 6000 system. Sequencing data were aligned to the mm10 reference genome using STAR in two-pass mode to output a BAM file sorted by coordinates. Mapped reads were assigned to annotated genes using featureCounts version 1.6.3, and differentially expressed genes were identified using DESeq2 version 1.30.1 with a 5% false discovery rate.

### DNA Extraction and Whole Genome Enzymatic Methylation Sequencing:

Genomic DNA was isolated from sorted cells using a DNA Extraction Kit (Qiagen) and sonicated to generate fragments of approximately 350 to 400 base pairs using S220 Focused Ultrasonicator (Covaris). Unmethylated cytosines were converted to uracils, and sequencing libraries were created using the NEBnext Enzymatic Methyl Seq Kit (New England Biolabs) according to the manufacturer’s instructions. DNA libraries were sequenced using an Illumina NovaSeq 6000 system following the manufacturer’s protocols.

### Whole Genome Enzymatic Methylation Sequencing Analysis:

Sequencing data quality was assessed using FastQC v0.11.4. Adapters were trimmed from the sequencing reads using Trim Galore! v0.4.4 using options (trim_galore -o $OUTDIR --fastqc --paired $FORWARD_READS $REVERSE_READS). Alignment to the mm10 reference genome was performed using Bismark v0.19.0 with options (bismark --multicore 6 --bowtie2 -N 1 $MM10 −1 $FORWARD_READS −2 $REVERSE_READS). Deduplication was performed with deduplicate_bismark (deduplicate_bismark -p -bam $BISMARK_ALIGNED_BAM). Library quality was assessed based on the percentage of reads that aligned to the genome. Library quality was considered sufficient if greater than 50% of reads uniquely aligned to the genome. Enzymatic methyl conversion efficiency was assessed by evaluating the percent of methylation observed in the CHH genome context. Enzymatic methyl conversion was considered sufficient when this value was less than 3%. Genome coverage was assessed using the bedtools genomecov software v2.25.0. Library genome coverage was considered sufficient if 80% of the genome had a depth of at least 10 reads. For each library that met these quality metrics, methylation percentages at individual CpG positions in the reference genome were quantified using the Bismark Methylation Extractor v0.19.0 program with options (bismark_methylation_extractor-p--comprehensive--bedgraph $BISMARK_DEDUPLICATED_BAM). DMRs among the datasets were detected using BSmooth DMR finder. DMRs have at least 10 CpGs and an absolute mean difference of >0.1. The following R packages were utilized for data analysis: Dplyr, Tidyverse, Pheatmap, and GenomicRanges. Visualization of CpG positions with at least 10× coverage on colored heatmaps (blue-white-red) reflects the percent methylated from 0 to 100%. Individual genomic loci were displayed using UCSC Genome Browser.

## Supplementary Material

Supplement 1 **Supplemental Figure 1.** Early decitabine treatment during T cell priming enhances germinal center Tfh differentiation.
(A-H) Pilot experiments to determine the optimal dose and timing of decitabine treatment. **(A)** Experimental schematic. (**B**) Percent and number of B cells, (**C**) CD8 T cells, (**D**) PD-1+ CD8 T cells, (**E**) CD4+ T cells, and (**F**) SMARTA T cells. (**G**) Percent and number of CXCR5+ Bcl6^HI^ GC Tfh cells, gated on SMARTA cells. (**H**) Bcl6 MFI of CXCR5+ Tfh cells. (I-M) Analysis of early SMARTA T cell differentiation in DAC or PBS treated recipients. (**I**) Experimental schematic. (**J**) Number of SMARTA cells. (**K**) Percent and number of early (CXCR5+ CD25−) Tfh and (CXCR5− CD25+) Th1 cells. (**L**) Bcl6 and (**M**) Blimp1 MFI of early Tfh and Th1 cells. (N) B6 mice were infected with LCMV and to analyze the polyclonal response following DAC treatment. Data are representative of a single independent experiment. * p<0.05, **p<0.01. Statistical significance was determined by a student’s t test.
**Supplemental Figure 2.** Dnmt3a restricts GC Tfh cell differentiation in a cell-intrinsic manner.
(A-B) WT or KO SMARTA mice were treated with tamoxifen to induce *Dnmt3a* deletion then transferred into B6 mice. Mice were infected with LCMV and CD4+ T cell responses were analyzed 7dpi. (**A**) Dnmt3a MFI gated on SMARTA T cells. (**B**) Quantitative PCR analysis of DNA from either WT or *Dnmt3a* cKO cells to determine the percent of SMARTA cells that had recombined at the *Dnmt3a* locus. (**C**) Percent IFNγ, TNFα, and IL-2 triple producers of SMARTA T cells. (D-E) Dnmt3a-Flox+, *ERT2-Cre*+, or *Dnmt3a-Flox*+ *ERT2-Cre*+ (cKO) SMARTA cells were adoptively transferred into B6 or Cre+ B6 mice following tamoxifen treatment. Recipients were infected with LCMV and the CD4+ T cell response was tracked in the blood. (**D**) Representative FACS plots of CD45.1 and CD4+ staining for detection of SMARTA T cells, gated on CD4+ T cells. The top row is from 7 dpi and the bottom is 30 dpi. (**E**) Quantification of the percent CD45.1+ of CD4+ T cells at 7, 15 and 30 dpi. (**F**) Experimental schematic. (**G**) Number of SMARTA cells in the spleen. (**H**) Quantification of the percent and number of CXCR5− Ly6C^HI^ mTh1, CXCR5+ Ly6C^HI^ mTfh, CXCR5+ Ly6C^LO^ mTfh, and CXCR5− Ly6C^LO^ double negative (DN) SMARTA cells. (**I**) Tcf1 MFI of SMARTA T cell subsets. (**J**) Tbet, Runx2 and Runx3 MFI of SMARTA T cell subsets. * p<0.05, **p<0.01. Statistical significance was determined by a student’s t test.
**Supplemental Figure 3.** Dnmt3a balances the plasticity and functionality of memory Tfh and Th1 cells.
(A-D) WT or KO memory SMARTA cells were generated following LCMV infection. At 60+ dpi, spleens were extracted and SMARTA cells were sorted into CXCR5− Ly6C^HI^ mTh1, CXCR5+ Ly6C^HI^ mTfh, and CXCR5+ Ly6C^LO^ mTfh populations, independently transferred into naïve recipients, and mice were infected with LCMV. (**A**) Purity analysis of FACS sorted
CXCR5− Ly6C^HI^ mTh1, CXCR5+ Ly6C^HI^ mTfh, and CXCR5+ Ly6C^LO^ mTfh cells. (**B**) Quantification of the numbers of SMARTA cells in the spleens across three repeat experiments. Quantifications of (**C**) Tbet MFI of CXCR5− Th1 cells, (**D**) IFNγ MFI of IFNγ+ cells, and (**E**) granzyme B MFI of CXCR5− Th1 cells. All data is from the spleen unless denoted as lung in the figure. (**F**) Bcl6 and (**G**) Tcf1 MFIs of CXCR5+ Tfh cells in the spleen. (H-N) WT or *Dnmt3* cKO memory SMARTA cells were generated following LCMV infection. At 60+ dpi, bulk SMARTA memory cells were FACS sorted and transferred into naïve recipients, and mice were infected with LCMV. (**H**) Experimental schematic. (**I**) Percent and number CXCR5+ Bcl6^HI^ GC Tfh SMARTA cells. (**J**) Quantification of Bcl6 MFI of CXCR5+ Tfh cells. (**K**) Percent and number of GC B cells. (**L**) Percent and number of CXCR5− Th1 cells. (**M**) Tbet MFI of CXCR5− Th1 cells. (**N**) Percent and number of IFNγ+ SMARTA cells. For A-G, data are representative of three independent experiments. For H-N, data are representative of a single independent experiment. * p<0.05, **p<0.01. Statistical significance was determined by a student’s t test.
**Supplemental Figure 4.** Dnmt3a silences genes associated with alternative T helper lineages in Tfh and Th1 cells.
(A-F) Whole genome enzymatic methylation sequencing was performed on WT and cKO Tfh and Th1 cells sorted at 7 days post LCMV infection. WT and cKO naïve CD4+ T cells were also analyzed as controls. Differentially methylated regions (DMRs) were called between WT and cKO cells of the same lineage. (**A**) A breakdown of DMRs by genomic location. (**B**) Enrichment analysis of H3K4me1 ChIP-seq peaks (GSM1694162) from naïve CD4+ T cells. (**C**) Transcription factor motif analysis of Tfh and Th1 DMRs. (**D**) Overlap analysis of Dnmt3a-dependnent DMRs and *de novo* DMRs, as identified in Figure 1. (E-F) UCSC genome browser snapshots of (**E**) *Bcl6*, and (**F**) *Runx2*. We also show tracks for ENCODE identified cis-candidate regulatory elements (cCREs) and mammalian conservation. Grey boxed identify hypomethylated DMRs. In the heatmaps below, select DMRs are shown. Each column represents a CpG site across the DMR. For the *Bcl6* locus only, we also show human CpG islands which were found using LiftOver, as well as a track containing CTCF ChIP seq peaks from Naïve CD4+ T cells (GSM3498276). (G-K) RNA sequencing was performed on WT and cKO Tfh and Th1 cells sorted at 7 days post LCMV infection. Differentially expressed genes (DEGs) were called between WT and cKO cells of the same lineage. (**G**) Quantification of DEGs, broken down by directionality. (**H**) Overlap analysis of upregulated DEGs that contain hypomethylated DMRs in Th1 cells. (**I**) *Lef1* transcript and protein analysis in effector WT and cKO Tfh/Th1 cells. (**J**) Overlap analysis of upregulated DEGs that contain hypomethylated DMRs in Tfh cells. (**K**) *Runx1* transcript and protein analysis in effector WT and cKO Tfh/Th1 cells. (L-N) Either WT, cKO, or DAC treated memory SMARTA cells were enriched and transferred into naïve B6 recipients and infected with LCMV. Analysis was performed at 3 or 7 dpi in the spleen. (**L**) Experimental schematic. (**M**) Blimp1 analysis at 3 dpi. (**N**) Foxo1, Runx2 and Runx1 analysis at 7 dpi. For L-N, data are representative of a single independent experiment. * p<0.05, **p<0.01, ***p<0.005. Statistical significance was determined by a one-way ANOVA.
**Supplemental Figure 5.** Early decitabine treatment partially inhibits Dnmt3a-mediated methylation programing at Tfh-associated genes.
(A, C, and E) UCSC genome browser snapshots of select DMRs. All shown DMRs are Dnmt3a-dependent DMRs (WT vs KO) that do not overlap with DAC-dependent DMRs. (B, D, and F) the mean difference in methylation (KO – WT or DAC – PBS) are shown across the select Dnmt3a-dependent DMR. (**A**) Dnmt3a-dependent DMR in Tfh cells at *Prdm1*, and (**B**) quantification of mean difference across the *Prdm1* DMR. (**C**) Dnmt3a-dependent DMR in Tfh cells at *Runx2*, and (**D**) quantification of mean difference across the *Runx2* DMR. (**E**) Dnmt3a-dependent DMR in Tfh cells at *Bcl6*, and (**F**) quantification of mean difference across the *Bcl6* DMR. (**G**) Heatmaps show mean methylation for all Dnmt3a-dependent DMRs associated with *Foxo1*, *Runx2, Runx3*, and *Id2*. (**H**) LCMV memory T cells (WT, cKO or DAC) were generated, CD4+ T cells were enriched, adoptively transferred into naïve B6 mice and infected with LCMV. MFI analysis of Tfh cells for Foxo1, Runx2, Runx3, and Id2. (**I**) Model. For all locus specific heatmaps, we also show tracks for ENCODE identified cis-candidate regulatory elements (cCREs) and mammalian conservation. Grey boxed identify DMRs, which are highlighted below. Each column represents a CpG site across the region. For A, data are representative of a single independent experiment. * p<0.05, **p<0.01, ***p<0.005, **** p<0.0001. Statistical significance was determined by a one-way ANOVA.

## ABBREVIATIONS

LCMV: Lymphocytic Choriomeningitis Virus
GC: germinal center
Th1: T helper 1
Tfh: T follicular helper
GC Tfh: germinal center T follicular helper
mTfh: memory Tfh
mTh1: memory Th1
Tem: T effector memory
Tcm: T central memory
dpi: days post infection
hpi: hours post infection
B6: C57BL/6J
1°: Primary
2°: Secondary
DMRs: differentially methylated regions
DEGs: differentially expressed genes
WGEM-seq: Whole genome enzymatic methyl sequencing
WGBS-seq: Whole genome bisulfite sequencing
cCRE: cis-candidate regulatory element
