# Supplementary material for: Dnmt3a-dependent de novo DNA methylation enforces lineage commitment and preserves functionality of memory Th1 and Tfh cells
Source: bioRxiv. 2025 Feb 15:2024.12.03.623450. Originally published 2024 Dec 6. Preprint. [Version 2] doi: 10.1101/2024.12.03.623450 (PMC11642886; doi:10.1101/2024.12.03.623450)
Supplement: Supplement 1 — Supplemental Figure 1. Early decitabine treatment during T cell priming enhances germinal center Tfh differentiation to primary and secondary viral infections. (A-C) Pilot experiment to determine the optimal dose and timing of decitabine treatment. Briefly, naïve SMARTA cells were transferred into B6 mice which were subsequently infected with LCMV. Mice were intraperitoneally injected with either PBS, a single dose of DAC at varying concentrations (0.35mg/kg, 0.75mg/kg, 1.5mg/kg) given 1 day post infection, or two doses of DAC (1.5mg/kg) given on days 1 and 2 post infection. Analysis was performed on the spleen at 7 days p.i. Data are representative of a single experiment. (A) Quantification of B cells, CD8 T cells, PD-1+ CD8 T cells and CD4+ T cells. (B) Quantification of the percent and numbers of SMARTA T cells and (C) CXCR5+ Bcl6HI GC Tfh cells, gated on SMARTA cells. Statistical significance was determined by a one way ANOVA (* p<0.05, **p<0.01, ***p<0.001). (D-E) Briefly, B6 mice which were infected with LCMV. Mice were intraperitoneally injected with either PBS or DAC (0.75mg/kg) at 20 hours post infection. Analysis was performed on the spleen at 7 days p.i. Data are representative of a single experiment. (D) Percent and number of PD-1hi GC Tfh cells, gated on CD44hi CD4+ T cells. (E) Percent and number of GC B cells, gated on total B cells. Statistical significance was determined by an unpaired t test (* p<0.05, **p<0.01, ***p<0.001). (F-H) B6 mice which were intranasally infected with Influenza PR8. Mice were injected with either PBS or DAC (0.35mg/kg) at 20 hours post infection. Analysis was performed on the mediastinal LNs and lung at 8 days post infection. Data are representative of a two independent experiment. (F) Quantification of NP-specific CD4+ T cells in the mediastinal LNs (mLNs) or lung. (G) Number of CXCR5- Th1 and CXCR5+ Tfh cells, gated on NP-specific CD4+ T cells. (H) Number of Foxp3+ regulatory T cells, gated on CD4+ T cells. Statistical si [file NIHPP2024.12.03.623450v2-supplement-1.pdf]

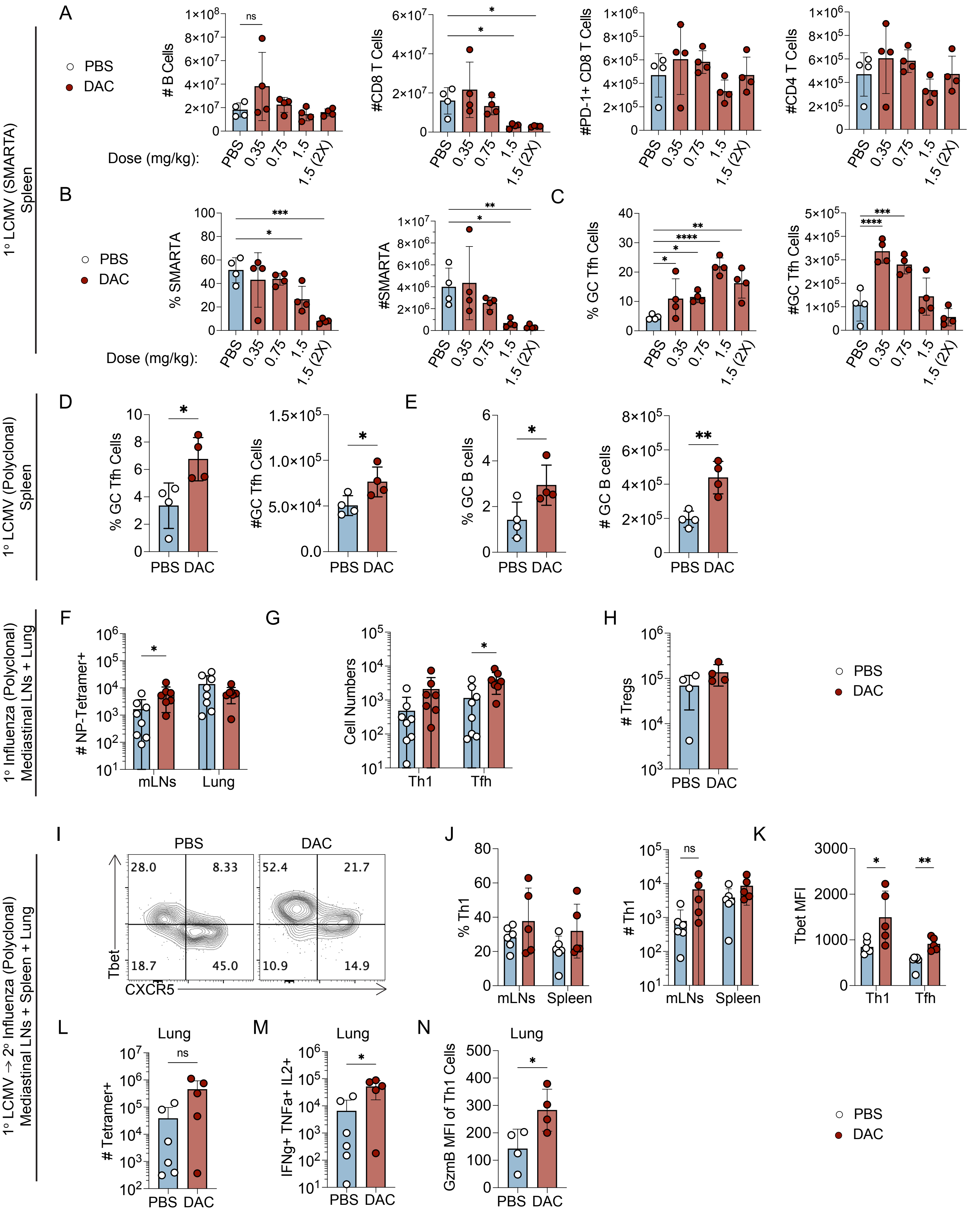

Supplemental Figure 1. Early decitabine treatment during T cell priming enhances germinal center Tfh differentiation to primary and secondary viral infections

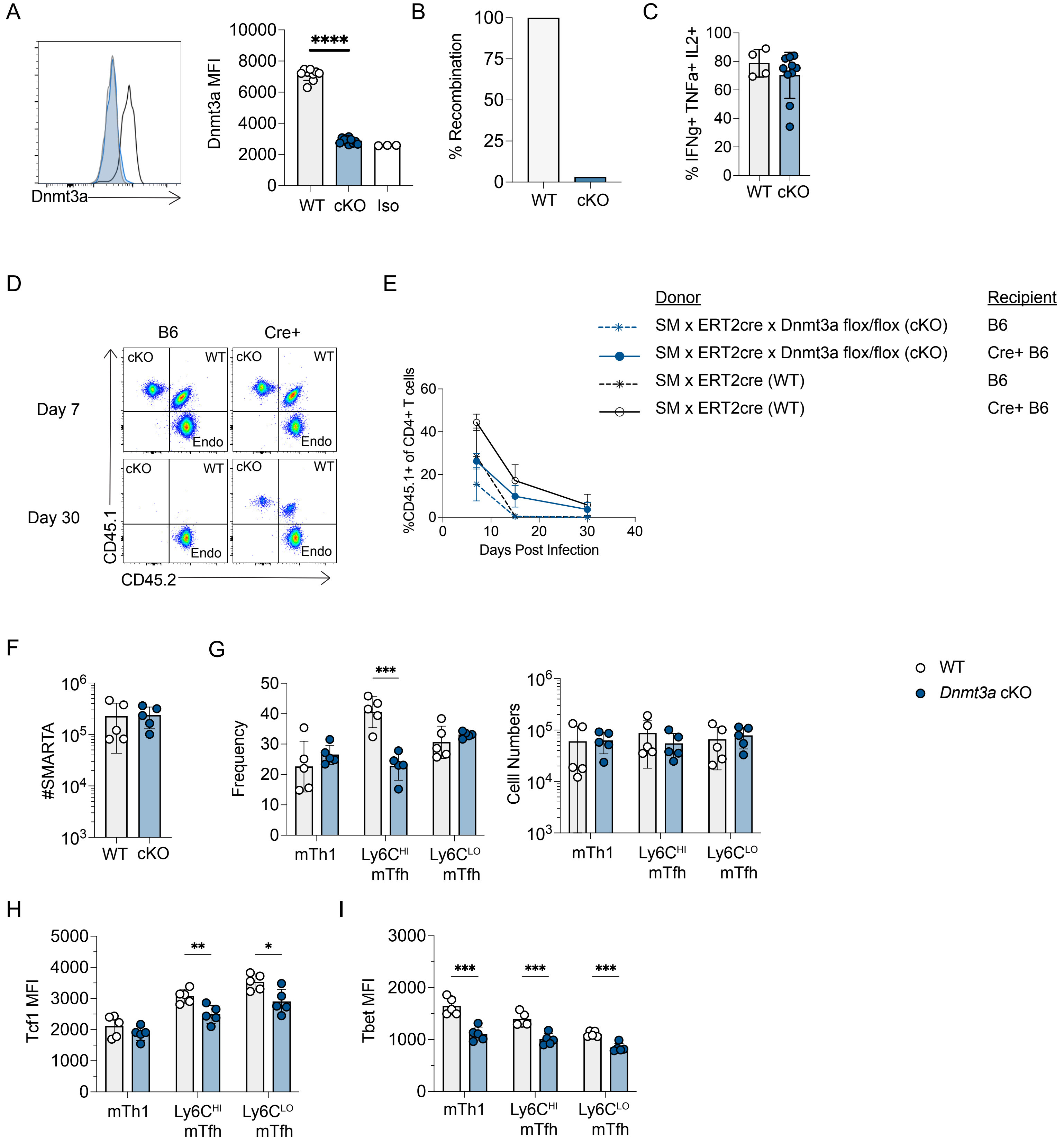

Supplemental Figure 2. Dnmt3a restricts GC Tfh cell differentiation in a cell intrinsic manner.

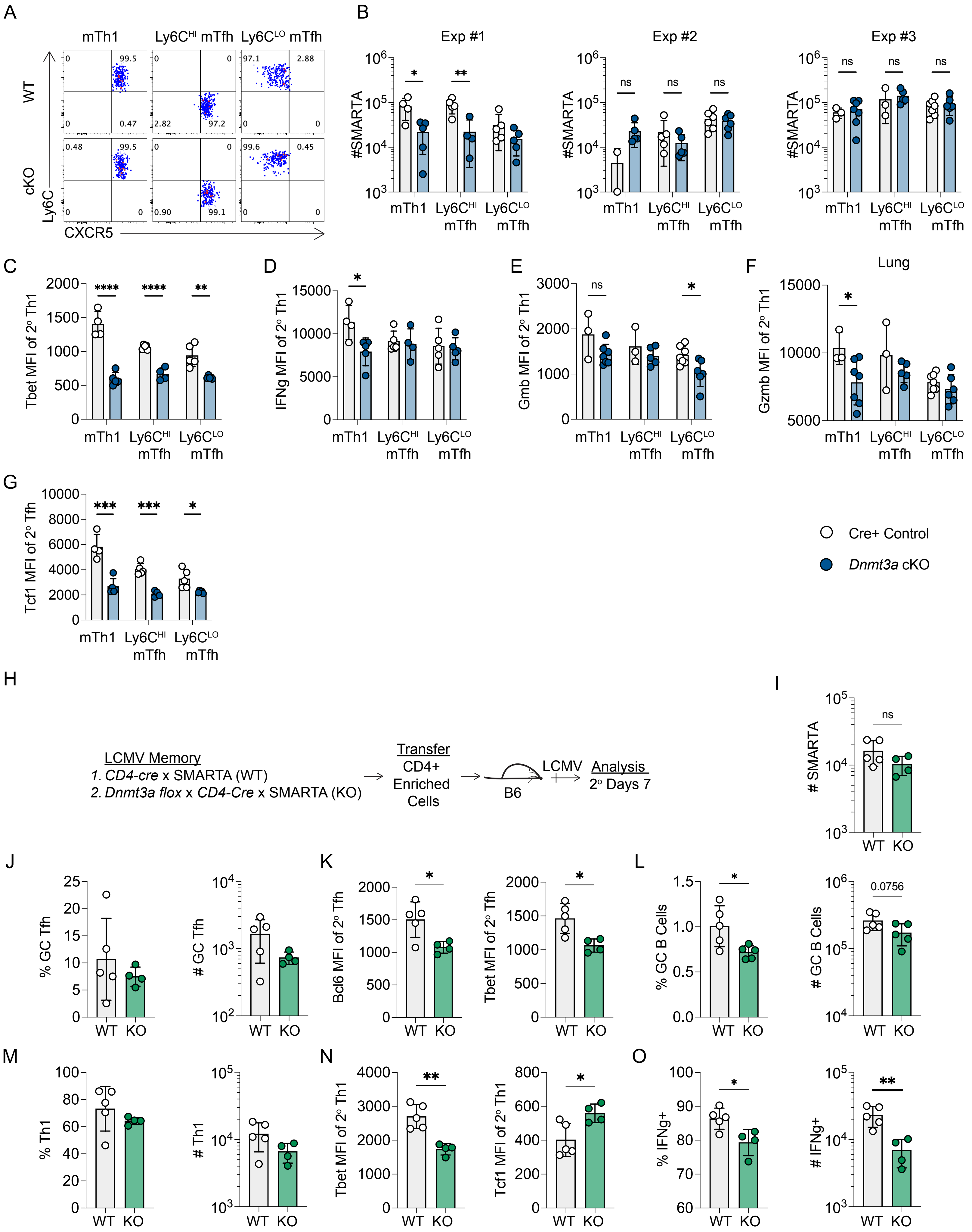

Supplemental Figure 3. Dnmt3a limits plasticity and preserves functionality of memory Tfh and Th1 cells

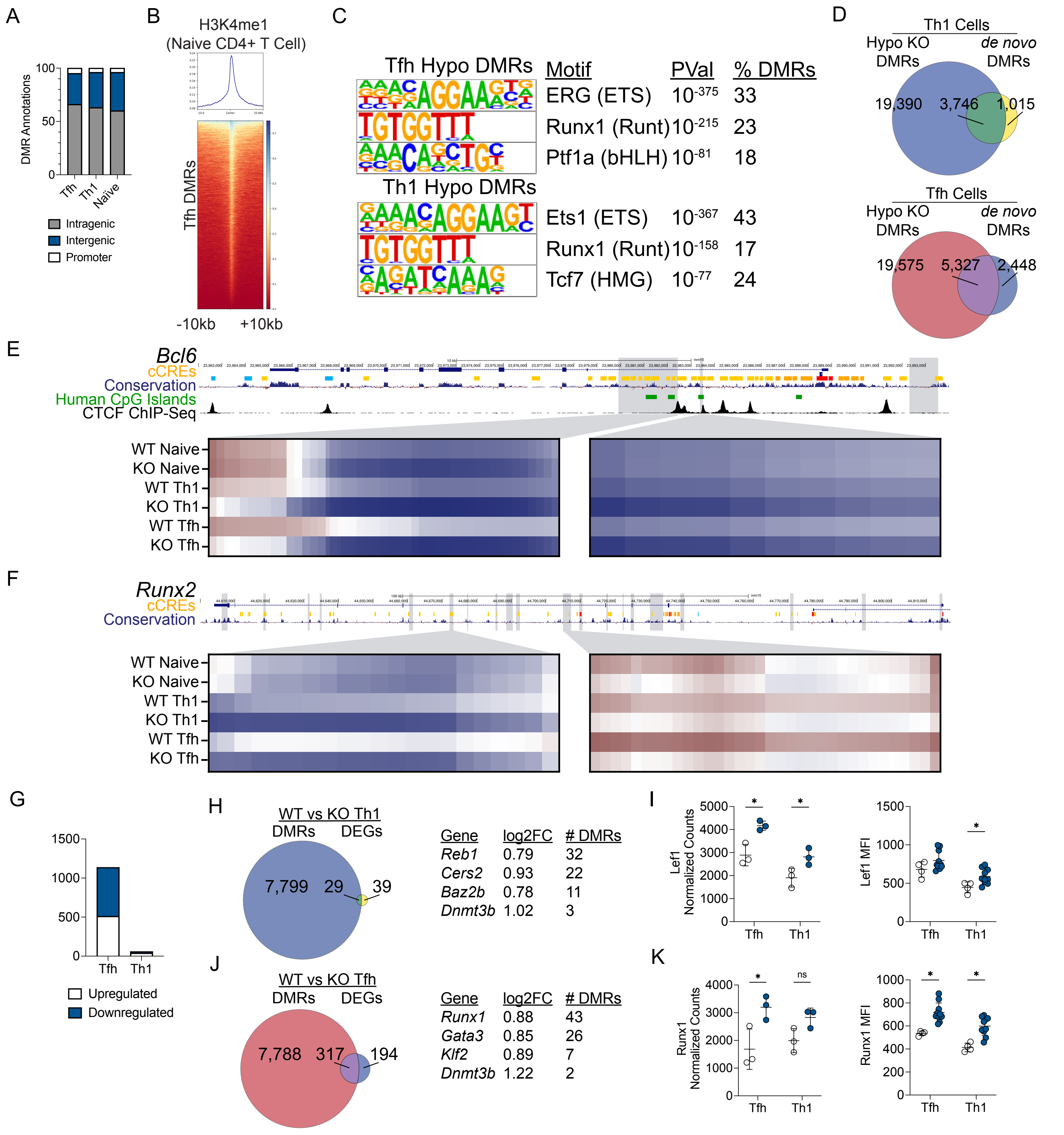

Supplemental Figure 4. Dnmt3a silences genes associated with alternative T helper lineages in Tfh and Th1 cells.

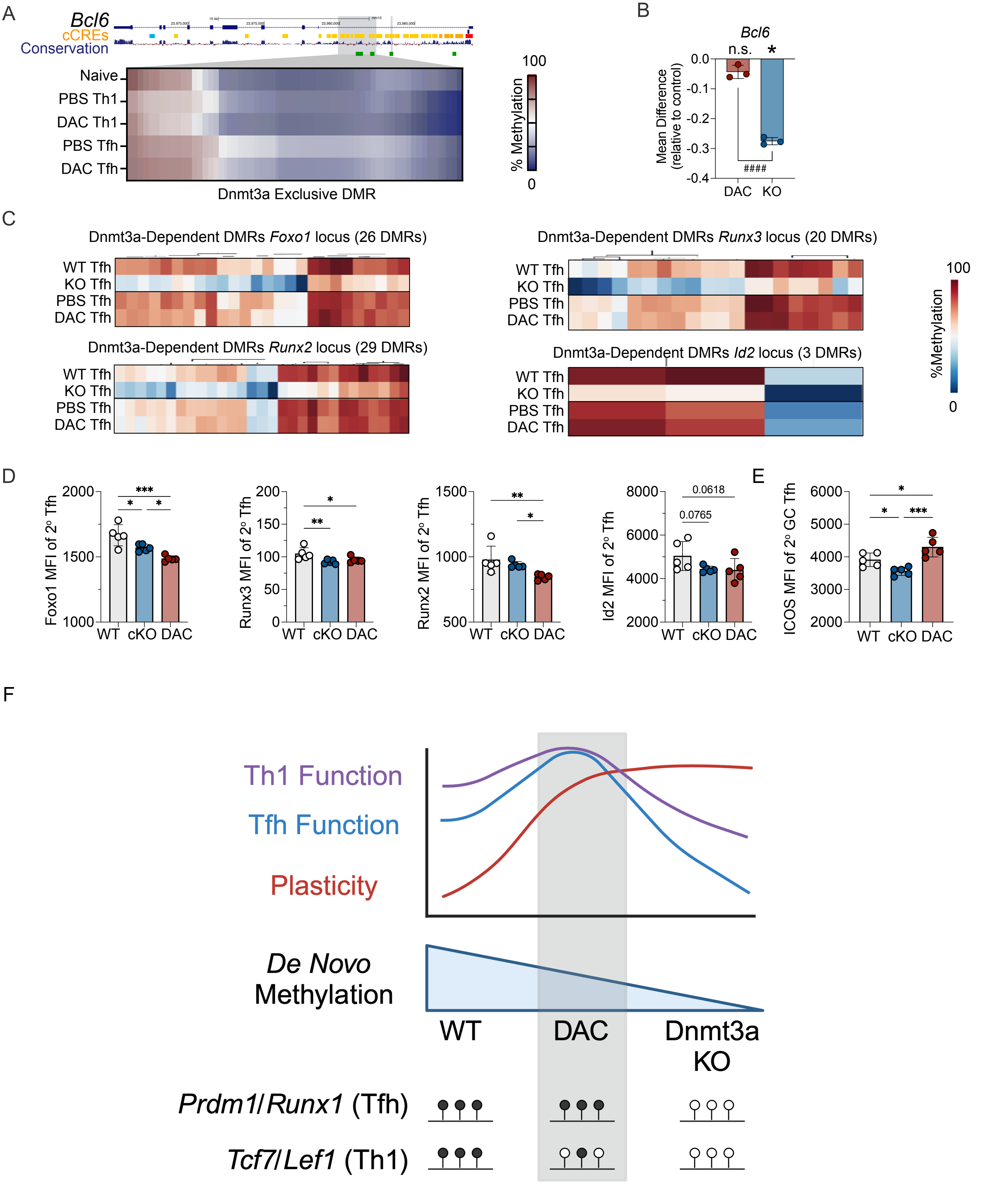

Supplemental Figure 5. *Dnmt3a* deficiency, but not early decitabine treatment, impairs silencing of the loci encoding Blimp1 and Runx1
