# Supplementary material for: Dnmt3a-mediated de novo methylation balances memory Th1 and Tfh cell plasticity and functionality
Source: bioRxiv. 2024 Dec 6:2024.12.03.623450. Preprint. [Version 1] doi: 10.1101/2024.12.03.623450 (PMC11642886; doi:10.1101/2024.12.03.623450)
Supplement: Supplementary file 1 [file media-1.pdf]

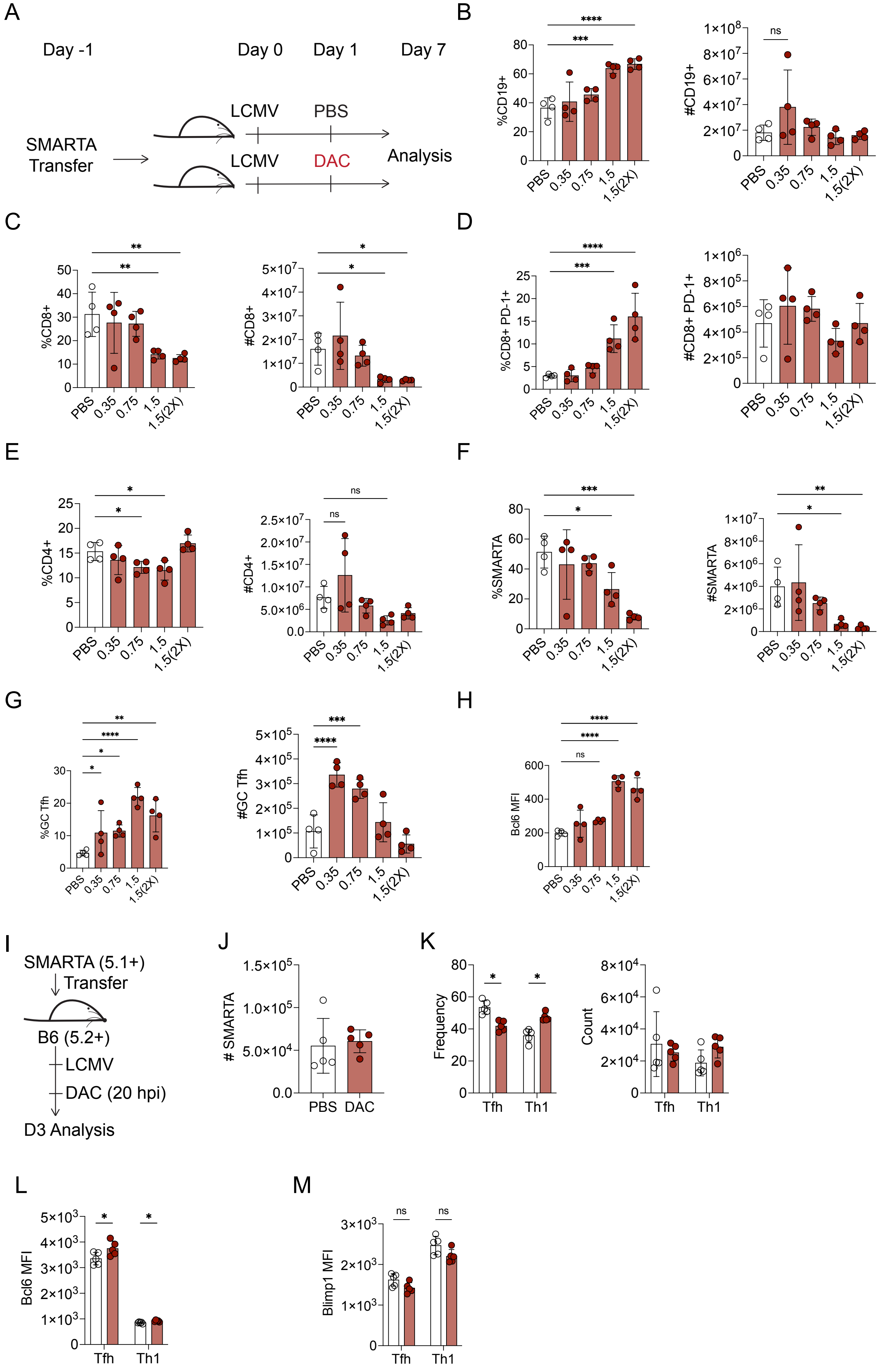

Supplemental Figure 1. Early decitabine treatment during T cell priming enhances germinal center Tfh differentiation.

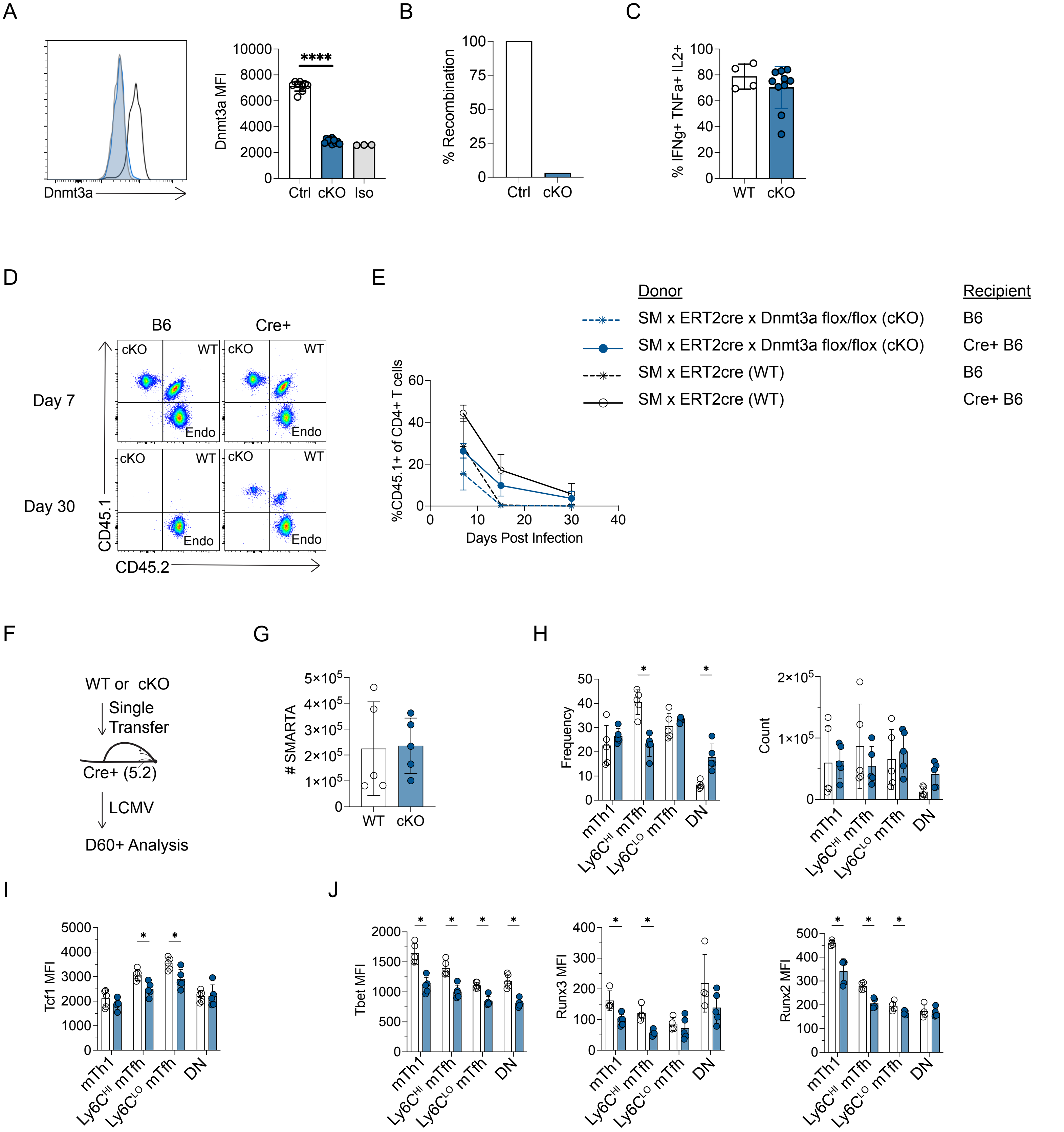

Supplemental Figure 2. Dnmt3a restricts GC Tfh cell differentiation in a cell intrinsic manner.

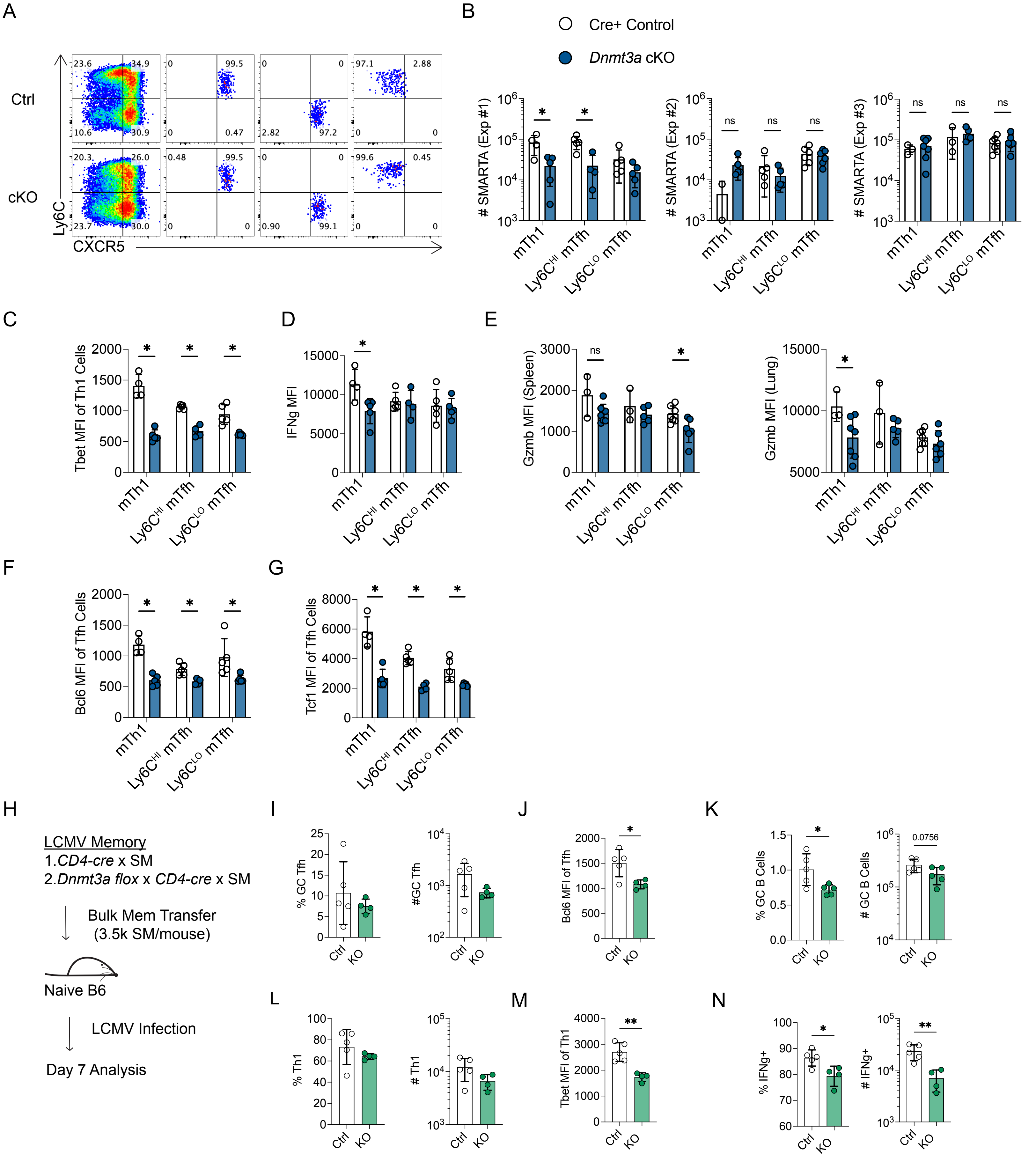

Supplemental Figure 3. Dnmt3a balances the plasticity and functionality of memory Tfh and Th1 cells.

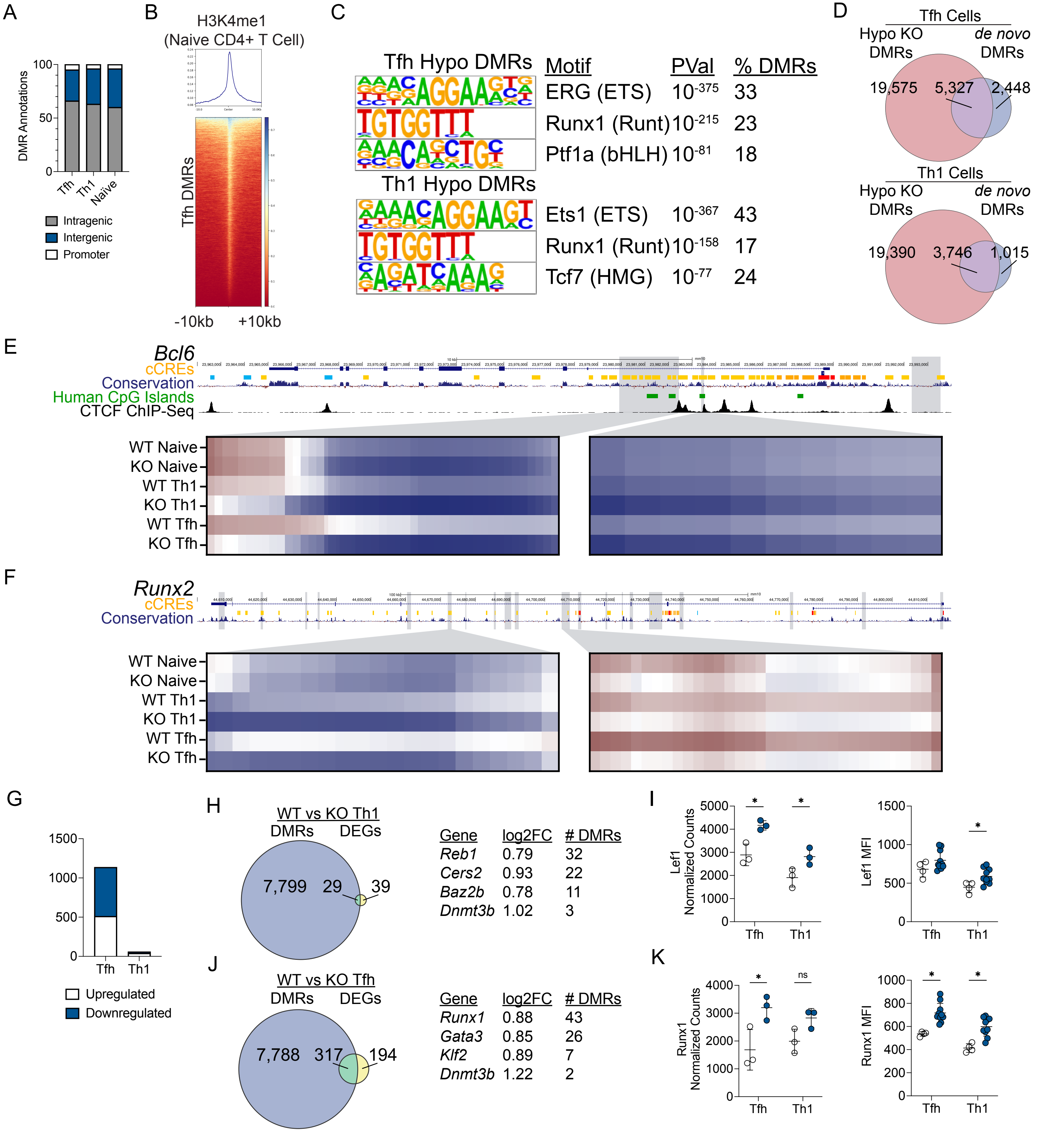

Supplemental Figure 4. Dnmt3a silences genes associated with alternative T helper lineages in Tfh and Th1 cells.

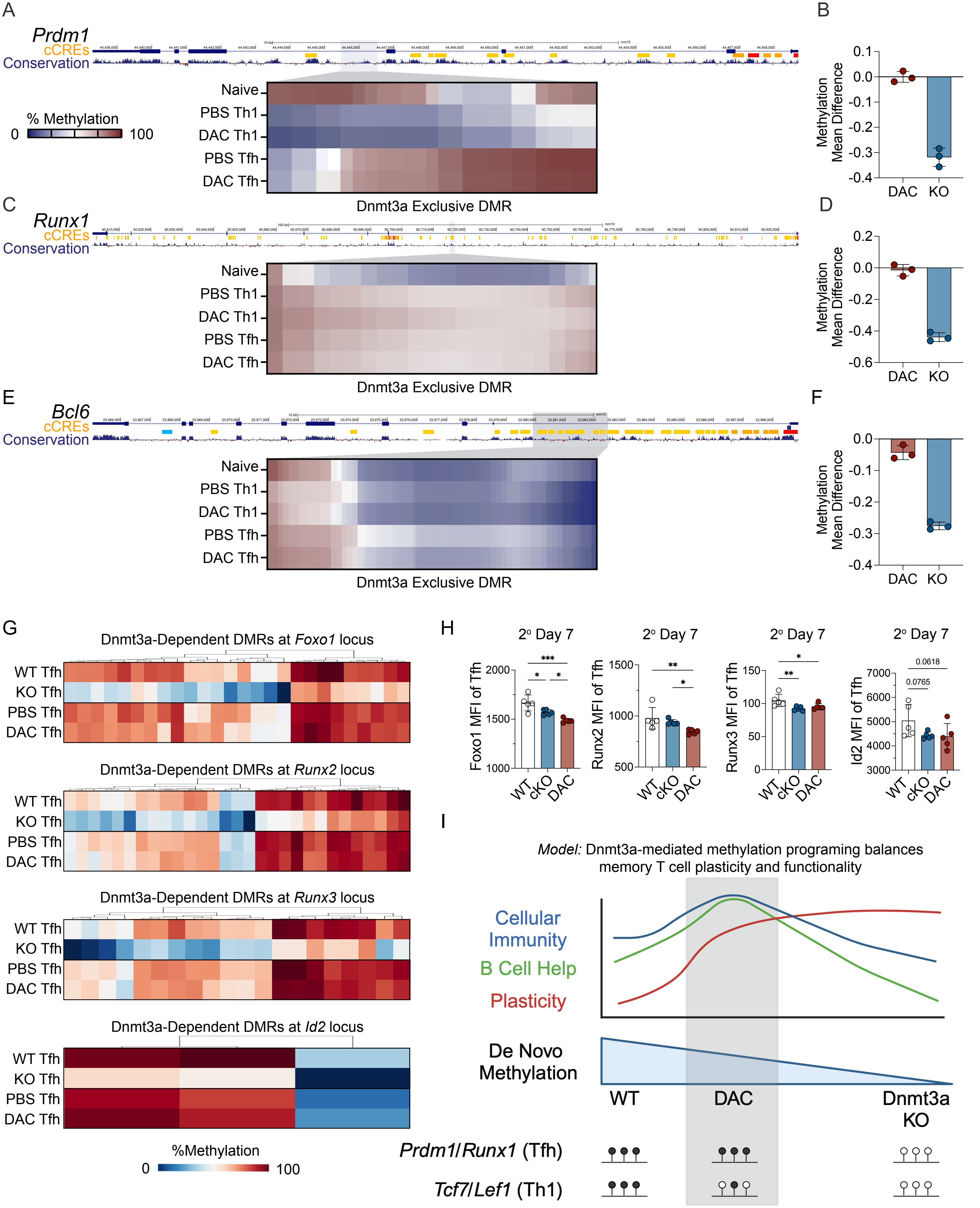

Supplemental Figure 5. Early decitabine treatment partially inhibits Dnmt3a-mediated methylation programing at Tfh-associated genes.
